# Phylogeny and evolution of plant macrophage migration inhibitory factor/D-dopachrome tautomerase-like proteins

**DOI:** 10.1186/s12862-015-0337-x

**Published:** 2015-04-14

**Authors:** Ralph Panstruga, Kira Baumgarten, Jürgen Bernhagen

**Affiliations:** RWTH Aachen University, Institute of Biology I, Unit of Plant Molecular Cell Biology, Worringerweg 1, 52074 Aachen, Germany; RWTH Aachen University, Institute of Biochemistry and Molecular Cell Biology, Pauwelsstrasse 30, 52074 Aachen, Germany

**Keywords:** *Arabidopsis thaliana*, Co-expression, D-dopachrome tautomerase, Macrophage migration inhibitory factor, Neofunctionalization, Oxidoreductase, Peroxisomes, Phylogeny

## Abstract

**Background:**

The human (*Homo sapiens*) chemokine-like protein macrophage migration inhibitory factor (*Hs*MIF) is a pivotal mediator of inflammatory, infectious and immune diseases including septic shock, colitis, malaria, rheumatoid arthritis, and atherosclerosis, as well as tumorigenesis. *Hs*MIF has been found to exhibit several sequential and three-dimensional sequence motifs that in addition to its receptor binding sites include catalytic sites for oxidoreductase and tautomerase activity, which provide this 12.5 kDa protein with a remarkable functional complexity. A human MIF paralog, D-dopachrome tautomerase (*Hs*DDT), has been identified, but its physiological relevance is incompletely understood. MIF/DDT-like proteins have been described in animals, protists and bacteria. Although based on sequence data banks the presence of MIF/DDT-like proteins has also been recognized in the model plant species *Arabidopsis thaliana*, details on these plant proteins have not been reported.

**Results:**

To broaden the understanding of the biological role of these proteins across kingdoms we performed a comprehensive *in silico* analysis of plant MIF/DDT-like (MDL) genes/proteins. We found that the *A. thaliana* genome harbors three *MDL* genes, of which two are chiefly constitutively expressed in aerial plant organs, while the third gene shows stress-inducible transcript accumulation. The product of the latter gene likely localizes to peroxisomes. Structure prediction suggests that all three Arabidopsis proteins resemble the secondary and tertiary structure of human MIF. MIF-like proteins are found in all species across the plant kingdom, with an increasing family complexity towards evolutionarily advanced plant taxa. Plant MDL proteins are predicted to lack oxidoreductase activity, but possibly share tautomerase activity with human MIF/DDT.

**Conclusions:**

Peroxisome localization seems to be a specific feature of a subset of MIF/DDT orthologs found in dicotyledonous plant species, which together with its stress-inducible gene expression might point to convergent evolution in higher plants and vertebrates towards neofunctionalization of MIF/MDL proteins in stress response pathways including innate immunity.

**Electronic supplementary material:**

The online version of this article (doi:10.1186/s12862-015-0337-x) contains supplementary material, which is available to authorized users.

## Background

In *Homo sapiens*, the protein family of chemotactic cytokines, i.e. chemokines, functions as traffic coordinators for the innate defense system, but it also orchestrates lymphocyte trafficking and homeostatic cell homing in the human body. Thus, chemokines represent a fundamental component in innate and adaptive immunity [1-9]. However, chemokines have also been subject to various mimicry mechanisms exploited by viruses and parasites. Macrophage migration inhibitory factor (MIF) is an evolutionarily ancient protein that is best known for its functions as an immune and inflammatory factor and that was more recently recognized to have chemokine-like properties to regulate a plethora of processes in the biology and pathophysiology of humans [10,11]. MIF is a prototypical member of the growing functional family of CLF (chemokine-like function) chemokines that share with classical chemokines chemokine receptor-mediated chemotaxis and cell recruitment activities, but that do not possess the canonical N-terminal cysteine residues or chemokine-fold [12-15]. Human MIF (*Hs*MIF) has 114 amino acids, forms a trimer in the X-ray structure [10,16], and is a required upstream component of human innate and adaptive immunity, but it is also overexpressed in various human diseases [10]. If dysregulated, *Hs*MIF is a pivotal mediator of inflammatory, infectious and immune diseases including septic shock, colitis, malaria, rheumatoid arthritis, and atherosclerosis, as well as in several tumors [14,17]. In fact, *Hs*MIF was discovered already 50 years ago, but it took until the early 1990s and mid-2000s until the MIF protein was characterized and the MIF receptors were identified, respectively [18-21]. MIF functions are mediated through three receptor proteins: on the one hand, MIF signals through the type II receptor protein CD74/invariant chain, but on the other hand it also serves as a non-cognate ligand for the classical CXC chemokine receptors CXCR2 and CXCR4 [19,20].

The structural interfaces governing MIF/receptor interactions have partly been unraveled, but important mechanistic details of its structure-activity characteristics, cell- and environment-specific receptor usage, cross-reactivity, interplay with *bona fide* ligands, or receptor complexes are unclear [14]. Moreover, MIF is abundantly expressed in the cytosol of numerous cells and features two evolutionarily conserved catalytic sites, i.e. a dopachrome tautomerase and a thiol-protein oxidoreductase (TPOR) activity, implying links to MIF’s role in cell cycle regulation [10,22]. The catalytic activities have been suggested to localize to a three-dimensional proline-2-containing tautomerase site at the N-terminus and a Cys-Xaa-Xaa-Cys motif-spanning sequence at amino acids 57–60, respectively. Both catalytic *Hs*MIF activities can be readily measured *in vitro*, but physiologic substrates have been elusive and the functional role of the activities *in vivo* is unclear. Strikingly, *Hs*MIF and its human paralog MIF-2/D-DT (D-dopachrome tautomerase, hereafter *Hs*DDT) are found across kingdoms with expression verified in mammals, *Xenopus laevis*, *Caenorhabditis elegans*, *Bifidobacterium longum*, *Clostridium acetobutylicum*, or unicellular parasites such as *Brugia malayi*. While co-expression of DDT and MIF has been observed in some species, functional evidence on DDT has been scarce. DDT shares with MIF an exacerbating role in endotoxic shock and models of melanoma, non-small cell lung carcinoma, and renal tumorigenesis, and mimics the cardioprotective effect of MIF in a mouse model of ischemia/reperfusion injury of the heart [23-26]. In contrast, the effects of DDT on cell survival and apoptosis are complex and the receptor mechanisms conveying DDT activity are unclear at the molecular level. Also, a combined functional analysis of both MIF proteins in other organisms has not yet been undertaken.

*Arabidopsis thaliana* is a dicotyledonous plant species and is arguably the best-studied model plant [27]. Although its genome sequence has been resolved more than a decade ago [28], approximately half of its ca. 30.000 genes remain functionally unknown or are annotated only on the basis of static analyses such as protein motifs or similarities [29]. The uncharacterized genes also include apparent *MIF*-like genes, whose existence in the Arabidopsis genome has been previously noted [30,31]. However, the genes have not yet attained any attention by the plant or cytokine/chemokine community, and accordingly neither the genes nor the respective gene products have been characterized to date.

To further broaden the understanding of the role of MIF-like proteins across kingdoms we performed a comprehensive *in silico* analysis of plant MIF/DDT-like (MDL) genes/proteins with an emphasis on the reference species *Arabidopsis thaliana*. We deployed several analysis tools to unravel the copy number, gene structure, expression profile and predicted subcellular localization of the Arabidopsis MDL genes/proteins. We extended the study by sequence alignment and phylogenetic analysis including a broad range of MDL sequences across the entire plant kingdom.

## Results

### *Arabidopsis thaliana* encodes three MIF-like proteins

**Figure 1:**
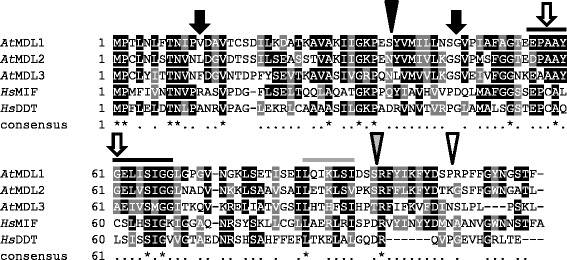
Multiple sequence alignment of *At*MDLs, *Hs*MIF and *Hs*DDT. Amino acid sequences of *At*MDL1 (AAO42959), *At*MDL2 (NP_195785), *At*MDL3 (AEE78824), *Hs*MIF (P14174) and *Hs*DDT (P30046) were aligned with ClustalW2 (http://www.ebi.ac.uk/Tools/msa/clustalw2/) using standard parameters. Subsequently, the alignment was shaded with BoxShade 3.21 (http://www.ch.embnet.org/software/BOX_form.html) using default settings. A black shade indicates identical amino acids; a grey shade denotes similar amino acids. In the consensus line, asterisks specify invariant amino acids and dots conserved amino acids with similar biophysical properties at a given position. The black triangle highlights the relative position of an intron found in all five genes, the white triangle the relative position of an intron present in *AtMDL1* and *AtMDL2* and the grey triangle the relative position of an intron found in *HsMIF* and *HsDDT*. The white arrows denote the position of C^57^ and C^60^ in *Hs*MIF, the black bar above part of the sequence indicates the location of InterProScan domain IPR019829 (MIF, conserved site) in *Hs*MIF and *Hs*DDT, which overlaps with the TPOR motif. The black arrows point at residues R^12^ and D^45^ in *Hs*MIF that form the pseudo-ELR motif and the grey bar above part of the sequence signifies the position of the predicted NES in *At*MDL1 and *Hs*MIF.

We performed BLASTP searches against the predicted proteome of the dicotyledonous reference species *Arabidopsis thaliana* (TAIR, http://www.arabidopsis.org/) using human MIF (GenBank accession number P14174) as a query sequence. This analysis revealed three hits with moderate sequence similarity to *Hs*MIF, comprising proteins At5g57170 (E value 3e-12, 33% identity), At5g01650 (E value 2e-12, 30% identity) and At3g51660 (E value 9e-7, 28% identity). The three proteins are of similar length as human MIF and DDT (115, 115 and 112 amino acids for the three Arabidopsis proteins versus 115 amino acids for *Hs*MIF and 118 for *Hs*DDT); accordingly, their calculated molecular weight is within a comparable range (ca. 11.3-12.7 kDa; Table 1). These values refer to the conceptual full-length proteins as predicted from the corresponding cDNA sequences. Human MIF and DDT are known to undergo proteolytic processing of the N-terminal methionine and thus in its mature form comprise only 114 and 117 amino acids, respectively [25,32]. The sequence similarity to human MIF and DDT extends nearly along the entire protein, with 14 invariant amino acid residues interspersed, except for the very C-terminus, which shows little sequence conservation between the five aligned proteins (Figure 1). The 14 invariant amino acids include a proline at position 2 (P^2^; note that numbering of amino acids in this study refers to the conceptual full-length sequences, disregarding the proteolytic processing of the N-terminal methionine), which is essential for the tautomerase activity of *Hs*MIF [33]. By contrast, the two cysteine residues (C^57^ and C^60^) that form the TPOR motif and are crucial for the oxidoreductase activity in *Hs*MIF [22,34] are not preserved in the Arabidopsis proteins (Figure 1). The Arabidopsis proteins also lack the so-called pseudo ELR motif (Figure 1). This sequence motif, which is comprised of the amino acids glutamate (E), leucine (L) and arginine (R), is found in a variety of chemokines. It is critical for receptor binding and essential for chemotactic activity of ELR^+^ CXC chemokines [35]. In *Hs*MIF, it is present in a non-canonical manner (‘pseudo’ ELR motif), constituted by non-adjacent residues in neighboring loops but with identical parallel spacing as in the authentic ELR motif [36].

**Table 1:** **Features of** ***At*** **MDL proteins in comparison to** ***Hs*** **MIF and** ***Hs*** **DDT**

	***Hs*** **MIF**	***Hs*** **DDT**	**At5g57170 (** ***At*** **MDL1)**	**At5g01650 (** ***At*** **MDL2)**	**At3g51660 (** ***At*** **MDL3)**
Number of amino acids	115	118	115	115	112
Molecular mass^a^	12476	12712	12324	12111	12210
Isoelectric point (pI)^a^	7.73	6.72	4.96	6.17	8.82
InterProScan domains	IPR001398	IPR001398	IPR001398	IPR001398	IPR001398
	IPR014347	IPR014347	IPR014347	IPR014347	IPR014347
	IPR019829	IPR019829			
Predicted localization signal	NES^b^		NES^b^		C-terminal PTS1^c^

^a^full-length protein based on calculation with ExPASy (http://web.expasy.org/compute_pi/); note that in most cases analyzed so far the N-terminal methionine is post-translationally removed by proteolytic processing).

^b^nuclear export sequence.

^c^peroxisome targeting sequence 1.

Analysis with InterProScan 5 (http://www.ebi.ac.uk/interpro/) indicated the presence of characteristic MIF (IPR001398) and tautomerase (IPR014347) domains as the sole recognizable features in the three Arabidopsis proteins (Table 1). Accordingly, we named these Arabidopsis MIF/DDT-like (MDL) polypeptides *At*MDL1 (At5g57170), *At*MDL2 (At5g01650) and *At*MDL3 (At3g51660), although the IPR019829 motif (MIF conserved site; consensus sequence [DE]-P-[CLV]-[APT]-x(3)-[LIVM]-x-S-[IS]-[GT]-x-[LIVM]-[GST]), which is located at amino acids 55–67 in *Hs*MIF and *Hs*DDT and corresponds to the catalytic TPOR site in *Hs*MIF, is missing in the three Arabidopsis proteins (Table 1, Figure 1). The three *At*MDL proteins exhibit considerable pairwise sequence similarity with *At*MDL1 and *At*MDL2, showing a higher degree of relatedness to each other than to *At*MDL3 (*At*MDL1-*At*MDL2: 60% identity, 75% similarity; *At*MDL1-*At*MDL3: 40% identity, 57% similarity; *At*MDL2-*At*MDL3: 50% identity, 69% similarity). This is somewhat higher than the kinship of *Hs*MIF and *Hs*DDT, which exhibit 37% identity and 52% similarity. Despite the high overall degree of sequence relationship, the calculated isoelectric point (pI) of the three Arabidopsis MDL proteins differs greatly, with a pI of 5.0 for *At*MDL1, 6.2 for *At*MDL2 and 8.8 for *At*MDL3, as compared to a pI of 7.7 for *Hs*MIF and 6.7 for *Hs*DDT (Table 1). The three Arabidopsis MDL proteins and their respective genes are largely uncharacterized since according to our literature searches no studies with functional data are available to date.

### Genomic organization and distribution of *AtMDL* genes

For *AtMDL1* and *AtMDL2*, the Arabidopsis reference database TAIR (The Arabidopsis Information Resource; http://www.arabidopsis.org/) lists two distinctive gene models each (At5g57170.1/At5g57170.2 and At5g01650.1/At5g01650.2, respectively). These models differ in the number of exons/introns and the localization of splice sites, resulting in different predicted transcripts, which suggests the potential occurrence of alternative splicing events at the transcript level. However, BLAST searches against Arabidopsis expressed sequence tags (ESTs) at the NCBI database solely support gene models At5g57170.1 and At5g01650.1, suggesting that the two other models (At5g57170.2 and At5g01650.2) may either rely on faulty genome annotation or may represent rare splicing variants. In the following, we therefore only considered the conceptual amino acid sequences that are based on gene models At5g57170.1 and At5g01650.1.

*AtMDL1* and *AtMDL2* are characterized by the presence of two introns, while *AtMDL3* has a single intron. The former is similar to the situation of *HsMIF*, which also harbors two introns, whereas the human *DDT* gene has three introns, of which the first is located outside the coding sequence in the 5´-untranslated region. Notably, the relative position of the first intron in At*MDL1*, *AtMDL2* and *HsMIF,* the second intron in *HsDDT* and the sole intron in *AtMDL3* is precisely preserved (Figure 1). This finding indicates common ancestry of the plant and human *MIF*/*DDT* genes and suggests that at least part of the genomic organization of the *MIF*/*DDT* genes has been retained since the separation of the plant and animal lineages ca. 1.6 billion years ago [37]. The relative positions of the second introns are also conserved for *AtMDL1* and *AtMDL2* on the one hand and *HsMIF* and *HsDDT* on the other hand, indicating the acquisition of lineage-specific introns later during evolution (Figure 1).

The *Arabidopsis thaliana* genome has been shaped by a whole genome duplication event, which resulted in extended yet reshuffled blocks of tandem repeated genomic regions that exhibit large-scale conservation in the number, order and orientation of genes [38]. Interrogation of the Plant Genome Duplication Database (http://chibba.agtec.uga.edu/duplication/) revealed that none of the *AtMDL* genes has a recognizable counterpart as the result of intragenomic duplication. This finding suggests that the diversification of the *AtMDL* genes occurred prior to the whole genome duplication event, which has been estimated to have occurred ca. 38 million years ago [39].

### Structure prediction of *At*MDL proteins

**Figure 2:**
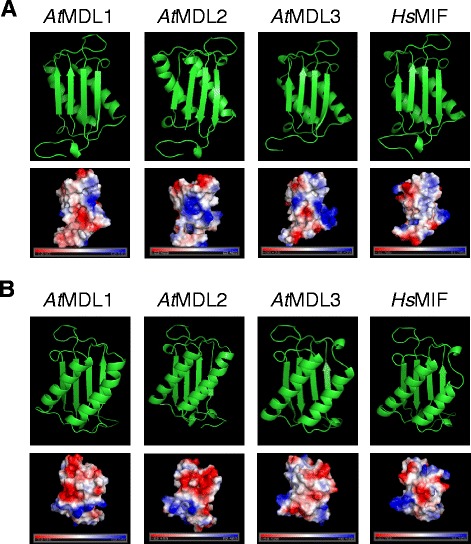
Predicted *At*MDL 3D structures. Amino acid sequences of the *At*MDL proteins were subjected to analysis *via* the PHYRE^2^ Fold Recognition server (http://www.sbg.bio.ic.ac.uk/phyre2/html/page.cgi?id=index) and rendered with PyMOL (http://www.pymol.org/). The predicted 3D structures (ribbon models and electrostatic surface potential) of *At*MDL1, *At*MDL2 and *At*MDL3 are visualized in comparison to the known X-ray-resolved 3D structure of *Hs*MIF. In the upper panels, green arrows symbolize β-sheets and green coils α-helices. In the lower panels, red color indicates an excess of negative and blue color an excess of positive charges near the surface, while white color specifies neutral regions. For simplicity, the structure of the monomer is shown only. **A** View with the four-stranded β-sheet in front. **B** View with the two antiparallel α-helices in front.

The three-dimensional (3D) structure of *Hs*MIF has been initially resolved by X-ray crystallography at 2.6 Å resolution [16]. *Hs*MIF crystallizes as a trimer, while solution analyses have produced variable results about the oligomerization state of *Hs*MIF, ranging from monomer to dimer or trimer species depending on the protein concentration and method applied [13]. The *Hs*MIF monomer consists of two antiparallel α-helices that pack against a four-stranded β-sheet. A 3D topology with similar structural elements was subsequently determined for *Hs*DDT on the basis of X-ray crystallography at a resolution of 1.54 Å [40]. We used the fold recognition server Phyre^2^ (Protein Homology/analogY Recognition Engine 2, http://www.sbg.bio.ic.ac.uk/phyre2/html/page.cgi?id=index) to predict *in silico* the 3D structure of the three *At*MDL monomers. This analysis, which is based on homology modelling, revealed a surprisingly well conserved tentative 3D structure of the Arabidopsis MDL proteins in comparison to *Hs*MIF (Figure 2), despite the limited similarity of these proteins at the level of the primary amino acid sequence (ca. 30% identity – see above). The noted architectural conservation is reminiscent of the high degree of 3D similarity between mammalian MIF/DDT proteins and tautomerase-active homologs found in protists (e.g. *Leishmania major* MIF, [41]) and bacteria (e.g. 4-oxalocrotonate tautomerase (4-OT) and 5-(carboxymethyl)-2-hydroxymuconate isomerase (CHMI; [33]). Further support for similar structures of *Hs*MIF and the *At*MDL proteins is provided by a second structure prediction algorithm (QUARK), which allows *ab initio* calculation of protein 3D structures. Although the 3D structure of *Hs*MIF monomer predicted with QUARK differs from the experimentally determined structure (in contrast to the X-ray based structure the two α-helices flank the β-sheets), QUARK calculated similar 3D structures for *Hs*MIF and the three *At*MDL proteins (Additional file 1: Figure S1). Together these findings further strengthen the notion that the *At*MDL proteins are *bona fide* co-orthologs of *Hs*MIF and *Hs*DDT that may share functional conservation with respect to core biochemical activities of this protein class. We further visualized the electrostatic surface potential of these proteins to find out whether the noted differences in the pI of MIF/MDL proteins (Table 1) is reflected by prominent alterations in surface charge distribution. Since this was not the case, we speculate that the amino acids causing the charge differences are either not surface-exposed and/or distributed to such an extent that differences become unrecognizable.

### Subcellular localization of *At*MDL proteins

Using a panel of prediction servers, we inspected the three *At*MDLs *in silico* for the presence of canonical targeting signals that could provide first hints on their subcellular localization. All three proteins lack a number of analyzed targeting signals for dedicated subcellular localization, including N-terminal secretion signals (SignalP 4.1, http://www.cbs.dtu.dk/services/SignalP/), transmembrane domains (TMHMM 2.0, http://www.cbs.dtu.dk/services/TMHMM/), transit peptides for mitochondrial (Mitoprot, http://ihg.gsf.de/ihg/mitoprot.html) or chloroplast (ChloroP 1.1, http://www.cbs.dtu.dk/services/ChloroP/) localization and nuclear import signals (NLStradamus, http://www.moseslab.csb.utoronto.ca/NLStradamus/). Lack of these sequence motif features is shared by *Hs*MIF and *Hs*DDT, which are secreted by non-conventional pathways that do not require ER/Golgi transit [42,43]. However, *At*MDL1 has a predicted leucine-rich nuclear export signal (NetNES 1.1, http://www.cbs.dtu.dk/services/NetNES/) at amino acid positions 85–91 and *At*MDL3 possesses a predicted C-terminal peroxisomal targeting sequence (PTS1 predictor; http://mendel.imp.ac.at/mendeljsp/sat/pts1/PTS1predictor.jsp) (Table 1). Notably, the predicted NES is shared by *Hs*MIF, where this putative signal occurs at the corresponding relative position (amino acid positions 83–90; Table 1).

Two of the three *At*MDLs have been previously identified in shot-gun proteomic studies of Arabidopsis organelles. *At*MDL1 was found as a stromal protein in the chloroplast proteome [44,45] and *At*MDL3 was discovered as a peroxisomal protein [46]. The latter localization is consistent with the presence of a C-terminal PTS1 localization signal in *At*MDL3 (Table 1) and also with fluorophore-based subcellular localization studies [47], whereas the experimentally found stromal localization of *At*MDL1 is not supported by a recognizable chloroplast-targeting sequence. Nevertheless, further experimental evidence is needed to substantiate the predicted subcellular localization of the *At*MDL proteins.

### Expression profiles of *AtMDL* genes

We used an Arabidopsis microarray-based gene expression analysis tool (Arabidopsis eFP Browser, http://bar.utoronto.ca/efp/cgi-bin/efpWeb.cgi) to analyze the expression profiles of the three *AtMDL* genes in different tissues, during development and upon different abiotic and biotic stress cues. We found that *AtMDL1* and in particular *AtMDL2* show fairly constitutive expression in most aerial plant tissues and during most developmental stages, whereas expression in roots is comparatively low. In contrast to this broad expression pattern, *AtMDL3* expression seems to be restricted to cotyledons, rosette leaves and sepals (i.e. all types of green leaves; Additional file 2: Figure S2A and Additional file 3: Table S1). Overall, *AtMDL1* and *AtMDL2* show higher basal transcript levels than *AtMDL3*, but reveal little alteration in transcript levels upon abiotic or biotic stress cues. By contrast, *AtMDL3* exhibits strongly enhanced transcript accumulation in leaves upon cold treatment (12 h at 4°C) and a less dramatic increase following osmotic stress, wounding and UV-B treatment (Additional file 2: Figure S2B and Additional file 3: Table S1). *AtMDL3* shows also elevated transcript levels in leaves upon exposure to various biotic stresses, including microbial elicitors (e.g. treatment with the peptide flg22) and pathogens (e.g. inoculation with *Botrytis cinerea*, *Pseudomonas syringae*, *Phytophthora infestans* or *Golovinomyces orontii*; Additional file 2: Figure S2C and Additional file 3: Table S1). Taken together, *AtMDL1* and *AtMDL2* reveal comparatively constant expression levels in most organs, tissues and conditions, whereas *AtMDL3* is characterized by lower basal levels, but elevated transcript accumulation in response to various abiotic and biotic stress cues.

### Co-expressed genes and interacting proteins

Co-expression of genes has recently been recognized as one possible predictor of gene products that cooperate in a given process or biochemical pathway [29,48,49]. Accordingly, a number of databases have been established to explore potential gene co-expression. We used the online tool ATTED-II (http://atted.jp/) to identify Arabidopsis genes that are co-expressed with the three *AtMDL* genes. For each gene we retrieved a list of the top 300 co-expressed genes and compared these for potential communalities. We noted that 67 of the 300 genes co-expressed with *AtMDL1* and *AtMDL2* are identical (Additional file 4: Table S2), suggesting that the two genes are largely expressed in the same conditions, together with a shared set of genes. Among these common genes is a conspicuously high number of genes coding for cytosolic or plastid-specific ribosomal proteins (23 in total). We subjected the co-expressed genes to PageMan-based overrepresentation analysis (http://mapman.mpimp-golm.mpg.de/general/ora/ora.shtml). This indeed revealed statistically highly significant (p < 1e-10) overrepresentation of genes encoding cytoplasmic (*AtMDL2*) and plastid-specific (*AtMDL1*) ribosomal proteins (Additional file 4: Table S2; sheet “overrepresented BINs”). Interestingly, human ribosomal protein RPS19 has been identified to interact with *Hs*MIF [50], suggesting that association with ribosomes and/or ribosomal proteins could be a general feature of MIF-like proteins. Notably, none of the genes that were found to be co-expressed with *AtMDL1* or *AtMDL2* is co-expressed with *AtMDL3* or *vice versa*, indicating that *At*MDL1 and *At*MDL2 on the one hand and *At*MDL3 on the other hand may function in fundamentally different biological processes. We noted the occurrence of several genes firmly implicated in plant defence among the top 300 genes co-expressed with *AtMDL3*, including *PEN2* [51], *PEN3* [52], *PAD4*/*SAG101* [53], *SID2* [54], *EFR* [55], *SOBIR1* [56], different *WRKY* genes [57], as well as *MLO2* and *MLO12* [58]. However, *AtMDL3* is not an integral part of the previously described defence regulon [48], although the genes of the defence regulon and the set of *AtMDL3*-co-expressed genes partially overlap (e.g. *MLO2*, *PEN2* and *PEN3*). In further support of a putative function in plant immunity, PageMan analysis indicated statistically highly significant (p < 1e-10) overrepresentation of receptor kinases among the genes co-expressed with *At*MDL3 (Additional file 4: Table S2; sheet “overrepresented BINs”). In sum, the results of the co-expression analysis are consistent with the expression profiles of the three *AtMDL* genes, which indicated mostly constitutive expression of *AtMDL1* and *AtMDL2* and stress-inducible expression of *AtMDL3* (see above).

We next interrogated the Plant Interactome Database (http://interactome.dfci.harvard.edu-/A_thaliana/) for potentially known physical interactors of the three *At*MDL proteins. This revealed one identified interactor each (on the basis of large-scale yeast two-hybrid screens; [59]) for *At*MDL1 and *At*MDL3. The protein interacting with *At*MDL1 is At2g47590 (photolyase/Blue Light Receptor 2/BLR2), a protein implicated in DNA repair, while *At*MDL3 interacted with At5g64160, a protein of unknown function. The biological relevance of these interaction partners identified by yeast-based methods remains, however, to be shown.

### MDL proteins in other plant species

To explore to which extent *MDL* genes are also present in other plant species, we performed BLAST searches with a focus on fully sequenced and annotated plant genomes at the Plant Genome Database (PlantGDB, http://www.plantgdb.org/prj/GenomeBrowser/). We included species that represent different levels of plant evolution, i.e. green algae (*Chlamydomonas reinhardtii* and *Volvox carteri*), a bryophyte (moss; *Physcomitrella patens*), a lycophyte (fern relative; *Selaginella moellendorffii*), a gymnosperm (*Picea sitchensis*; Sitka spruce) and various angiosperms (monocotyledonous and dicotyledonous plant species). In some cases we found MDL proteins with otherwise identical amino acid sequences but differing C-termini in the database. These likely comprise annotated splice variants of the same gene locus, as reported above for *At*MDL1 and *At*MDL2 (see above). For consistency, in these cases we only included the variants that seemingly resemble the *At*MDL versions, although we cannot rule out that some of the putative splice variants might be biologically meaningful. Similarly, we removed variants that are nearly identical and possibly just the result of sequencing errors or natural genetic variation within a species.

We discovered that each species harbors genes encoding MDL proteins, with typically multiple copies (paralogs) present per species, except the non-vascular plants (green algae and the moss), which encode a single MDF protein each (Table 2). We also noted a trend towards a higher number of MDL paralogs in dicotyledonous than in monocotyledonous plant species, with an average of 3.2 paralogs per dicotyledonous species and no species having less than three paralogs, compared to two paralogs per monocotyledonous species.

**Table 2:** **Plant MDL proteins**

	**Scientific name**	**Common name**	**Number of MDL proteins**	**Number of MDL proteins with PTS1-like motif**
Green algae	*Chlamydomonas reinhardtii*	n.a. ^a^	1	0
Green algae	*Volvox carteri*	n.a.	1	0
Moss	*Physcomitrella patens*	Spreading-leaved earth moss	1	0
Lycophyte (fern relative)	*Selaginella moellendorffii*	Spike moss	4	0
Gymnosperm	*Picea sitchensis*	Sitka spruce	2	0
Monocotyledonous angiosperms	*Brachypodium distachion*	Purple false brome	2	0
	*Hordeum vulgare*	Barley	2	0
	*Oryza sativa*	Rice	2	0
	*Sorghum bicolor*	Sorghum	2	0
	*Zea mays*	Maize	2	0
Dicotyledonous angiosperms	*Arabidopsis thaliana*	Thale cress	3	1
	*Glycine max*	Soybean	4	1
	*Lotus japonicus*	Birdsfoot trefoil	3	1
	*Prunus persica*	Peach	3	1
	*Solanum lycopersicon*	Tomato	3	1
	*Vitis vinifera*	Grapevine	3	1

^a^n.a., not applicable.

Using CLUSTALW2 we generated a multiple sequence alignment of the curated set of plant MDL proteins identified in the context of our BLAST searches (Additional file 5: Figure S3). This analysis revealed seven invariant amino acids (corresponding to M^1^, P^2^, N^9^, P^35^, A^59^, G^67^ and R^95^ in the *At*MDLs) plus a number of highly conserved residues. Each of the invariant residues is also present in *Hs*MIF and *Hs*DDT (Figure 1), suggesting that these are crucial amino acids for the structure and/or function of MDL proteins. Overall, there is good conservation of amino acid sequences along the entire plant proteins, except at the very C-terminus. As expected, the angiosperm MDL sequences showed the highest level of conservation, while the sequences of the more distantly related gymnosperms, the lycophyte *S. moellendorffii* and the non-vascular plants (green algae and moss) were more variable.

The multiple sequence alignment revealed that most plant MDL proteins harbor either one or no cysteine residue. Exceptions are the algal MDL sequences of *C. reinhardtii* and *V. carteri*, which include two cysteine residues, and one of the *S. moellendorfii* sequences with three cysteines (Additional file 6: Figure S4). If present, the cysteine residues are in positions different from those seen in *Hs*MIF. Thus, the vast majority of plant MDL proteins lack the capacity to form an intramolecular disulphide bridge.

We recognized the occurrence of potential variants of the C-terminal PTS1 peroxisome localization signal in some of the sequences. Closer inspection of these amino acid sequences with PTS1 predictor (http://mendel.imp.ac.at/mendeljsp/sat/pts1/PTS1predictor.jsp) indeed corroborated the presence of likely PTS1 sequences in most of these proteins, while prediction results were ambiguous for two of the analyzed polypeptides (*G. max* ACU19241 and *L. japonicus* AFK35159). These findings suggest that not only *At*MDL3, but also MDL proteins of other plant species localize to peroxisomes. We noted that each dicotyledonous plant seems to encode one putatively peroxisome-localized MDL paralog, while these were apparently absent from monocotyledonous plants and outside the angiosperm lineage (Additional file 5: Figure S3). This notion was further substantiated by dedicated BLAST searches using *At*MDL3 as a query sequence, which revealed that PTS1-carrying MDL sequences appear to be restricted to dicotyledonous plant species. A basic isoelectric point as recognized for *At*MDL3 (Table 1) seems to be a common feature of these MDL proteins, as they all exhibit calculated pI values of 8.8-9.2.

Unlike the predicted PTS1 signal in *At*MDL3, which could be also recognized in several paralogs from other plant species, the predicted NES in *At*MDL1 could not be confirmed in the *At*MDL1 relatives. Although all amino acid sequences from this clade show a tendency towards an NES in the respective region (corresponding to amino acid 85–91 in *At*MDL1), the threshold for a positive prediction is only passed in the case of *At*MDL1 (owing to the presence of an isoleucine in position 87). The relevance of these *in silico* analyses remains to be tested experimentally.

### Phylogenetic analysis of plant MDL proteins

**Figure 3:**
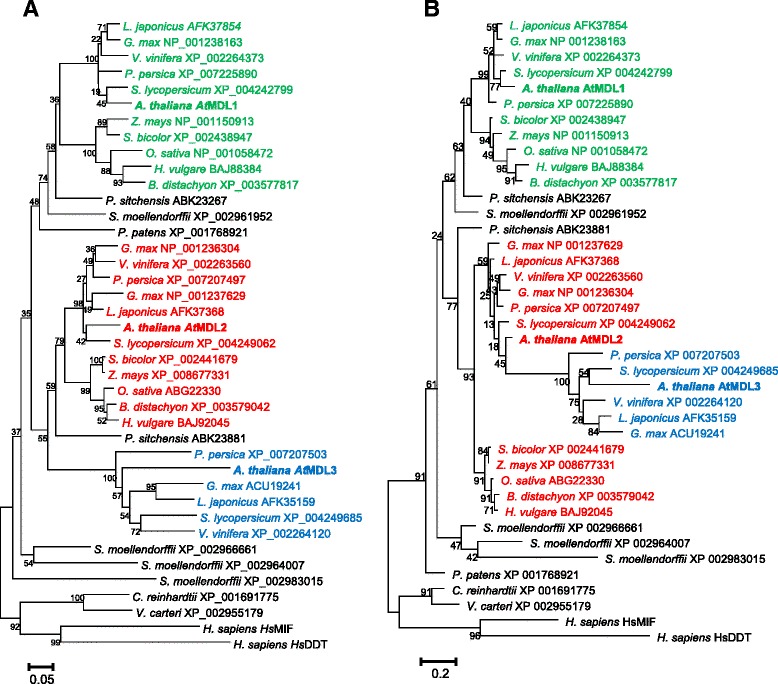
Phylogenetic analysis of plant MDL sequences. The evolutionary history of MDL proteins was inferred using either the Neighbor-Joining method (**A**; [105]) or the Maximum Likelihood method based on the JTT matrix-based model (**B**; [106]). The analysis involved 40 MDL sequences. All ambiguous positions were removed for each sequence pair. There were a total of 131 positions in the final dataset. Evolutionary analyses were conducted in MEGA5 [104]. *At*MDL1, *At*MDL2 and *At*MDL3 are shown in bold. Angiosperm MDL proteins grouping with the Arabidopsis proteins are coloured in green, red and blue, respectively. **A** The optimal tree with the sum of branch length = 4.91136864 is shown. The percentage of replicate trees in which the associated taxa clustered together in the bootstrap test (100 replicates) is shown next to the branches [107]. The tree is drawn to scale, with branch lengths in the same units as those of the evolutionary distances used to infer the phylogenetic tree. The evolutionary distances were computed using the p-distance method [108] and are in the units of the number of amino acid differences per site. **B** The tree with the highest log likelihood (−3971.5772) is shown. The percentage of trees in which the associated taxa clustered together is shown next to the branches. Initial tree(s) for the heuristic search were obtained automatically as follows. When the number of common sites was < 100 or less than one fourth of the total number of sites, the maximum parsimony method was used; otherwise BIONJ method with MCL distance matrix was used. The tree is drawn to scale, with branch lengths measured in the number of substitutions per site.

We extended our study by performing phylogenetic analysis of the subset of plant MDL protein sequences applying two widely used computational phylogenetic methods (Neighbor-Joining and Maximum Likelihood). Results of these analyses consistently revealed that *At*MDL1 is a member of a clade that comprises sequences from both mono- and dicotyledonous plant species (Figure 3; see also Additional file 7: File S2 for Newick notation of the phylogenetic trees). By contrast, results differ for *At*MDL2. While the distance matrix-based Neighbor-Joining method indicates that the *At*MDL2 clade also harbors sequences from both mono- and dicotyledonous plant species (Figure 3A), the probability-based Maximum Likelihood tree separates MDL members from these two lineages (Figure 3B). In both phylogenetic trees the two sequences of the gymnosperm plant *P. sitchensis* each group as sister branches of these major clades, suggesting that the respective dichotomous split occurred before the gymnosperm-angiosperm separation ca. 285 million years ago [60]. In the Neighbor-Joining tree, the moss (*P. patens*) MDL sequence groups together with the *At*MDL1 clade and one of the four lycophyte (*S. moellendorffii*) sequences, suggesting that this branch reflects the most ancestral MDLs of land plants. This issue remains, however, unresolved in the Maximum Likelihood tree.

*At*MDL3 is a member of a discrete clade, containing exclusively sequences of dicotyledonous MDL proteins with a predicted C-terminal peroxisome localization signal. This clade is a sister clade of the *At*MDL2-containing clade, suggesting that the *At*MDL3 lineage originates from a gene duplication event, involving a common progenitor of *At*MDL2 and *At*MDL3, after the divergence of monocotyledonous and dicotyledonous plant species ca. 145 million years ago [61]. As expected, human *Hs*MIF and *Hs*DDT cluster as out-groups in both trees and show closest relationship to MDL sequences from primitive plants such as green algae and three out of the four lycophyte MDLs.

Based on the phylogenetic separation we established dedicated clade-specific sequence alignments of *At*MDL1-, *At*MDL2- and *At*MDL3-like angiosperm MDL proteins (Additional file 8: Figure S5). This revealed that *At*MDL2-like sequences show the highest degree of sequence conservation, with 72 invariant amino acids within this clade, as compared to 56 and 46 invariant amino acids in *At*MDL1- and *At*MDL3-like sequences, respectively.

## Discussion

Our *in silico* analysis revealed that plant MDL proteins resemble *Hs*MIF and *Hs*DDT with regard to primary amino acid sequence and predicted three-dimensional structure (Figures 1 and 2). Together with the presence of this type of protein in other kingdoms of life (e.g. in protists [41] and eubacteria [62]), this finding suggests a common evolutionarily preserved core biochemical function for MDL proteins. Human MIF is a multifunctional polypeptide with at least three biological activities. First, it serves a role as an extracellular cytokine/chemokine that binds to the HLA class II histocompatibility antigen gamma chain (also known as the cell surface receptor CD74) or to the classical CXC chemokine receptors CXCR2 and CXCR4, thereby modulating innate immune responses [14,19,20]. The significance of a more broad relationship of *Hs*MIF to classical chemokine receptors was most recently corroborated by the discovery of *Hs*MIF/CXCR7 interactions on platelets [63]. Of note, this suggests that CXCR7 shares with CXCR4 not only the cognate ligand CXCL12, but also interacts with *Hs*MIF, [14]. In addition, *Hs*MIF has two physically separated catalytic sites that confer tautomerase as well as oxidoreductase activity, although the authentic *in vivo* substrates of these enzymatic activities remain unknown [34,64]. The contribution of these catalytic activities to *Hs*MIF´s chemokine function is controversially discussed and has not been fully resolved [10,13,65,66].

Plant MDL proteins seem to lack at least two of these three roles, namely chemokine function and oxidoreductase activity. Unlike vertebrates, plants do not have professional mobile immune cells such as macrophages. Instead each plant cell largely relies on its own capacity to combat microbial intruders (cell-autonomous immunity; [67,68]). Consistent with this type of innate immunity, plants do not possess components such as chemokines that could attract migrating immune cells to biotic stress sites. Plant MDL proteins are therefore unlikely to be secreted as signaling molecules to the extracellular space. This notion is supported by the absence of a recognizable N-terminal secretion signal and the lack of the so-called pseudo-ELR motif. Lack of a canonical N-terminal secretion signal in *Hs*MIF in conjunction with experimental findings has led to the view that human MIF and DDT are secreted *via* unconventional secretion pathways [42,43,69]. It nevertheless remains at least a formal possibility that also plant MDL proteins exert an extracellular function. In favor of this latter notion would be the finding that *At*MDL3 and related plant MDLs exhibit a fairly basic pI value above 8 (Table 1). *Hs*MIF has a pI value of 7.73 and it is believed that this feature contributes to endothelial deposition of extracellular MIF, similar to the well-known arrest chemokines [20]. The some 50 classical chemokines generally exhibit basic pI values in the range between 7.5 and 8.8 and it is this biophysical property that guarantees their endothelial immobilization following secretion on the extensively acidic proteoglycan (“glycocalix”) layer of the endothelium. Once immobilized, chemokines form a haptotactic gradient enabling them to mediate leukocyte arrest and transmigration during homeostatic homing processes or in inflammatory leukocyte extravasation [70,71]. In fact, acidic proteoglycans have been proposed to function as co-receptors in leukocyte arrest [72,73]. Biochemical evidence for direct MIF-proteoglycan interaction is still missing, but both human and mouse MIF are known to be deposited on the endothelial surface following secretion from endothelial cells and/or leukocytes and thrombocytes, promoting leukocyte activation and arrest [20,74]. Moreover, the surface form of CD74/invariant chain, a receptor for human and mouse MIF, is chondroitin sulfate-modified, a posttranslational prerequisite necessary for high affinity interaction with MIF [75].

Two closely linked cysteine residues form the central part of the TPOR site that is required for oxidoreductase activity in *Hs*MIF (Figure. 1; [22,34]). These paired cysteines are thought to serve a thioredoxin-like role during redox reactions catalyzed by *Hs*MIF. To also exert such an enzymatic activity the presence of paired cysteine residues would be expected in plant MDL proteins. However, most plant MDLs either have none or only one cysteine residue, with only few plant MDLs harboring two or more cysteines (Additional file 6: Figure S4). Exceptions are the algal MIFs encoded by the *C. reinhardtii* and *V. carteri* genomes, which each possess two similarly spaced cysteines in the same relative position (Additional file 6: Figure S4). Based on these amino acid features it is conceivable that at least the majority of plant MDLs do not possess oxidoreductase activity. This is in agreement with MIF-like proteins from several other non-vertebrate species, which were also suggested to lack this catalytic capacity [41,62,76]. However, it seems that plant MDLs have the principal potential to share tautomerase activity with their counterparts from other kingdoms of life (animals and protists). Evidence for this assumption is conservation of an N-terminal proline residue that is known to form the catalytic site for tautomerase activity (Figure 1). Since to our knowledge this sequence feature is present in all MIF proteins known so far, tautomerase activity could be the ancestral function of MDLs. Although no *in vivo* tautomerase substrate has been identified, the presumed conservation of tautomerase activity may hint at a chemical or chemical class whose presence is conserved across a broad range of taxa. D-dopachrome has been recognized as an artificial substrate of *Hs*MIF and other MDLs [42]. This compound is a cyclization product of L-3,4-dihydroxyphenylalanine (also known as L-DOPA) and an intermediate in the biosynthesis of melanin-type pigments. L-DOPA is best known for its role as a precursor molecule of various human neurotransmitters, but it also exists in plants where it seems to serve a role as precursor of different secondary plant metabolites [77]. Although plants lack conventional melanins, they are able to synthesize catechol melanin, which is also chemically related to L-DOPA [78]. It remains to be seen whether the natural substrate of MDL´s tautomerase activity is indeed related to L-DOPA.

We observed a tendency towards an increased copy number of *MDL* genes with increasing organismal complexity within the plant lineage. While evolutionarily older non-vascular plants such as algae and mosses contain only one *MDL* copy, evolutionarily younger vascular plants contain two or more paralogs (Table 2). A further increase in complexity of the *MDL* gene family can be seen within the angiosperm clade: While monocotyledonous plant species typically have two *MDL* copies, dicotyledonous species encode three or more MDL paralogs (Table 2). The additional copy in dicotyledonous plant species represents a type of MDL with unique gene and protein features. This MDL type, in Arabidopsis represented by *At*MDL3, has a C-terminal peroxisome targeting sequence (PTS1) and a different overall amino acid composition, as reflected by a markedly higher isoelectric point and a separate position within the phylogenetic tree (Tables 1 and 2, Figure 3). Moreover, the respective gene has at least in Arabidopsis a different genomic organization (lacking one intron; Figure 1), is the only Arabidopsis *MDL* that shows stimulus-dependent expression, and is co-expressed with a unique gene set (Additional file 4: Table S2). Together, these features point to neofunctionalization of this plant MDL paralog in dicotyledonous plants.

The Arabidopsis product of this MDL paralog (*At*MDL3) is predicted (by presence of the C-terminal PTS1 sequence; Table 1) and has been experimentally shown (by proteomic and cell biological analysis; [46,47]) to localize to peroxisomes. Plant peroxisomes are involved in numerous biological processes, including primary and secondary metabolism, development, as well as responses to biotic and abiotic stress cues. Similar to peroxisomes in mammalian cells, they are best known for their role in fatty acid oxidation (β-oxidation). In plants, they are also involved in the biosynthesis of the phytohormones indole acetic acid (IAA, an auxin) and jasmonic acid and they contribute to the process of photorespiration [79]. Transcript accumulation of *AtMDL3* is responsive to various abiotic and biotic stress stimuli, indicating that the respective protein may play a role in these conditions. Indeed, peroxisomes have been found to be responsive to abiotic stress [80,81] and biotic stress [82,83]. With regard to the latter, PEN2, an atypical myrosinase presumably involved in the biosynthesis of toxic glucosinolate metabolites, has been found to be associated with peroxisomes [51,84]. Notably, *PEN2*, which is also part of an antifungal defence regulon [48], is in the list of genes co-expressed with *AtMDL3* (Additional file 4: Table S2). Accordingly, *At*MDL3 may have a role in pathogen defence. If this was true, and if this was a general feature of angiosperm *At*MDL3-like proteins, then we may face the situation of convergent evolution in higher plants and mammals/vertebrates, characterized by neofunctionalization of MIFs/MDLs towards a role in innate immunity.

The remaining two types of plant *MDLs* (represented by *AtMDL1* and *AtMDL2*) show constitutive expression and are both co-expressed with an overlapping set of genes. This essentially resembles the expression profile of *HsMIF*, which was also described to be constitutively expressed in immune cells and stress-responsive tissues of the endocrine system [10]. Among the genes co-expressed with *AtMDL1* and *AtMDL2* are a remarkably high number of genes encoding ribosomal proteins. Notably, *Hs*MIF has been reported to interact with ribosomal protein S19 (RPS19), thereby attenuating its pro-inflammatory function [50]. As RPS19 can be released in inflammatory lesions by apoptotic cells, it has been suggested to act as an extracellular negative regulator of MIF function in this context [50]. Co-expression with a greater number of genes coding for ribosomal proteins suggests that at least *At*MDL1 and *At*MDL2 may act together with ribosomes, e.g. during protein biosynthesis. The similar expression pattern and an overlapping set of co-expressed genes further indicate that the two genes might be functionally redundant in Arabidopsis. Owing to a lack of dedicated targeting sequences, one might expect that the *At*MDL1 and *At*MDL2 proteins localize to the cytoplasm and/or the nucleus (because of passive diffusion). The functional relevance of a putative NES detected in *At*MDL1 awaits experimental verification.

Only two proteins have so far been identified as interaction partners of Arabidopsis MDLs. According to large-scale yeast two-hybrid data *At*MDL1 interacts with At2g47590 (photolyase/Blue Light Receptor 2/BLR2) and *At*MDL3 interacts with At5g64160 (a protein implicated in DNA repair; [59]). None of these interactions has been validated *in planta* so far. In contrast to the *At*MDL proteins, a substantial number of interacting proteins have been identified for *Hs*MIF. These comprise PAG [85], JAB/CSN5 [86], BNIPL [87], HPO [88] and RPS19 ([50]; see also above). Since currently no common theme or interrelation of these interaction partners can be recognized, one may speculate that *Hs*MIF functions through a number interactions with a diverse set of proteins.

To further unravel the function of plant MDLs, genetic, molecular and cell biological analyses will be necessary. T-DNA insertion lines for all three *AtMDL* genes can be found in public repositories (http://signal.salk.edu/cgi-bin/tdnaexpress) and could be used to identify potential phenotypes of *AtMDL* knock-out plants. Further protein-protein interaction screens are required to uncover additional interaction partners of the Arabidopsis MDL proteins. Moreover, *in planta* assays will be necessary to validate these yeast-based interactors. Subcellular localization studies using fluorophore-tagged proteins will shed light on the cellular site of action of the Arabidopsis MDLs. Analysis of plant MDLs may help to finally get a hold of the long-sought after natural substrates of the MIF/DDT tautomerase activities.

## Conclusions

We performed an in depth *in silico* analysis of plant MDL proteins. This unraveled that MIF/DDT-like proteins are present in all plant taxa, with an increasing number of paralogs in higher plant species. Plant MDLs share extensive sequence similarity (Figure 1) and predicted 3D structure (Figure 2) with their non-plant counterparts. They are predicted to lack oxidoreductase activity but possibly retained tautomerase activity as catalytic function. Phylogenetic analysis (Figure 3) allowed reconstruction and time estimates of the likely phylogeny of the MDL protein family in the course of plant evolution. The genome of the dicotyledonous model plant species *Arabidopsis thaliana* harbors three *MDL* genes, of which two are constitutively expressed and share an overlapping set of co-expressed genes, many of which code for ribosomal proteins. The third gene exhibits stress-inducible transcript accumulation and is co-expressed with a number of genes implicated in plant immunity. This gene encodes an MDL variant (*At*MDL3) with a C-terminal peroxisomal targeting signal (PTS1). The (co-) expression pattern and subcellular localization of *At*MDL3 suggest convergent evolution in higher plants and vertebrates towards MDL variants with a novel role in innate immunity. Precedence for such a scenario is provided by the convergent neofunctionalization of xanthine dehydrogenase towards aldehyde oxidase after recurring gene duplication events [89]. Functional analyses will be required to validate this hypothetical convergent neofunctionalization of MDL proteins in two separate kingdoms of life.

The comprehensive characterization of plant MDL proteins as undertaken in this study should aid in understanding the broader biological role of these proteins across kingdoms and should form the basis for future experimental work on these proteins. The catalytic activities of MIF have been extensively studied by numerous laboratories across the globe for more than 15 years. However, most of these studies have focused on the pure *in vitro* activity. Moreover, the physiologically relevant substrates have remained elusive and the functional role of these activities in MIF’s physiologic and pathophysiologic activities in humans and rodents is still unclear and under debate. Studying the catalytic activities of MDL proteins in plants and identifying novel MDL interaction partners could thus aid in uncovering the long sought-after natural substrates.

A broad body of evidence now clearly suggests that *Hs*MIF is an evolutionarily conserved protein that has both intra- and extracellular functions. In the human and mouse studies, the extracellular activities of MIF have been primarily addressed, with mostly only indirect evidence obtained about its intracellular effects. We therefore hypothesize that clarifying MIF’s role in a remote organism/kingdom such as plants in which a circulation/extracellular space-based mobile immune and defense system is missing, could provide valuable novel information about MIF’s intracellular effects, assuming that these are highly conserved and likely appeared first in the evolution of MIF proteins.

## Methods

### Sequences and BLAST analysis

The amino acid sequences used in this study were retrieved from NCBI/GenBank and can be found in Additional file 9: File S1. BLAST searches were performed against the NCBI (http://blast.st-va.ncbi.nlm.nih.gov/Blast.cgi), TAIR (http://www.arabidopsis.org/Blast/index.jsp) and Plant Genome Database (PlantGDB; http://www.plantgdb.org/prj/GenomeBrowser/), respectively. *AtMDL* gene models were retrieved from TAIR (http://www.arabidopsis.org/).

### Multiple sequence alignments

Multiple amino acid sequence alignments were established with CLUSTALW2, a general purpose DNA/protein multiple sequence alignment program (http://www.ebi.ac.uk/Tools/msa/clustalw2/; [90]) using standard parameters. Shading of alignments for conserved amino acids was performed with the help of BoxShade 3.21 (http://www.ch.embnet.org/software/BOX_form.html).

### Structure prediction and visualization

Structure prediction was carried out with the Phyre^2^ fold recognition server (http://www.sbg.bio.ic.ac.uk/phyre2/html/page.cgi?id=index; [91]) and the QUARK server (http://zhanglab.ccmb.med.umich.edu/QUARK/; [92]) using standard parameters. Phyre^2^ uses homology modeling based on the alignment of hidden Markov models. It also incorporates *ab initio* folding simulation to model regions of proteins with no detectable homology to known structures. QUARK models are built from small fragments (1–20 residues long) by replica-exchange Monte Carlo simulation under the guide of an atomic-level knowledge-based force field. Calculated 3D structures were visualized with PyMOL (http://www.pymol.org/; The PyMOL Molecular Graphics System, Version 1.7.4 Schrödinger, LLC), either as a cartoon showing the secondary structures (α-helices and β-sheets) or as a space-filling model revealing the electrostatic surface potential. The latter is calculated by averaging charges over a small region of space using a quasi-Coulombic-shaped convolution function (charge-smoothed potential).

### Online tools

Protein parameters such as molecular mass and isoelectric point were calculated with ExPASy bioinformatics resource portal (http://www.expasy.org/). The presence of signal peptides and transmembrane domains was explored with SignalP 4.1 (neural network-based prediction of the presence and location of signal peptide cleavage sites in amino acid sequences from different organisms; http://www.cbs.dtu.dk/services/SignalP/; [93]) and TMHMM 2.0 (hidden Markov model-based prediction of the presence and location of transmembrane helices in proteins; http://www.cbs.dtu.dk/services/TMHMM/; [94]), respectively. Subcellular protein localization was analyzed *via* Mitoprot (prediction of mitochondrial targeting sequences; http://ihg.gsf.de/ihg/mitoprot.html; [95]), ChloroP 1.1 (neural network-based prediction of chloroplast transit peptides; http://www.cbs.dtu.dk/services/ChloroP/; [96]), NLStradamus (hidden Markov model-based prediction of nulear localization signals; http://www.moseslab.csb.utoronto.ca/NLStradamus/; [97]) NetNES 1.1 (a combined neural network and hidden Markov model-based prediction of nuclear export sequences; http://www.cbs.dtu.dk/services/NetNES/; [98]) and PTS1 predictor (prediction of peroxisomal targeting sequence 1; http://mendel.imp.ac.at/mendeljsp/sat/pts1/PTS1predictor.jsp; [99]).

Searches for protein domains were performed with InterProScan 5, which scans for matches against the InterPro collection of protein signature databases (http://www.ebi.ac.uk/Tools/pfa/iprscan5/; [100]). Arabidopsis gene expression data were retrieved *via* the Arabidopsis eFP browser, a pictographic exploration tool for microarray data (http://bar.utoronto.ca/efp/cgi-bin/efpWeb.cgi; [101]). Co-expression analysis was performed using ATTED-II, a database that uses the weighted Pearson´s correlation coefficient to compare microarray-based gene expression profiles to reveal co-expression (http://atted.jp/; [102]). Arabidopsis protein-protein interaction data were obtained from the Plant Interactome Database (http://interactome.dfci.harvard.edu/A_thaliana/), which represents a repository of large-scale yeast two-hybrid results. Overrepresentation of co-expressed genes was analyzed with the ORA feature of the PageMan tool, which allows effective comparative analysis of multiple microarray experiments (http://mapman.mpimp-golm.mpg.de/general/ora/ora.shtml; [103]) using the TAIR8 Arabidopsis protein set as a control group.

### Phylogenetic analysis

The evolutionary history of plant MDL proteins was reconstructed with Neighbor-Joining and Maximum Likelihood analysis on the basis of CLUSTALW-aligned MIF/MDL sequences using MEGA5 software [104]. For the Neighbor-Joining tree, the p-distance amino acid substitution model was applied, using uniform rates among sites and pairwise deletion. For the Maximum Likelihood tree, the Jones-Thornton-Taylor (JTT) model was applied, using uniform rates and all sites. The Nearest Neighborhood-Interchange (NNI) method was employed for inference of the Maximum Likelihood tree. Both procedures (Neighbor-Joining and Maximum Likelihood) use the bootstrap method with 100 replicates for testing the phylogeny.

## Additional files

Additional file 1: Figure S1.
*Ab initio* prediction of *Hs*MIF/*At*MDL 3D structures with QUARK. Amino acid sequences of the *At*MDLs were subjected to analysis *via* the QUARK 3D structure prediction server (http://zhanglab.ccmb.med.umich.edu/QUARK/) and rendered with PyMOL (http://www.pymol.org/). The predicted 3D structures (ribbon models) of *At*MDL1, *At*MDL2 and *At*MDL3 are visualized in comparison to the predicted structure of *Hs*MIF. Additional file 2: Figure S2.
*AtMDL* expression profiles. Gene expression data for *AtMDL1*, *AtMDL2* and *AtMDL3* are shown according to the Arabidopsis eFP browser and represent data sets from development (A), abiotic (B) and biotic (C) stress. Additional file 3: Table S1.
*AtMDL* expression profiles. Gene expression data for *AtMDL1*, *AtMDL2* and *AtMDL3* are shown according to the Arabidopsis eFP browser (http://bar.utoronto.ca/efp/cgi-bin/efpWeb.cgi) and represent data sets from development, abiotic and biotic stress. Additional file 4: Table S2.
*AtMDL* co-expression. Sheet “AtMDL1”: Top 300 genes co-expressed with *At*MDL1 according to ATTED-II ((http://atted.jp/). Highlighted in red are those genes that overlap with genes that are co-expressed with *At*MDL2. Sheet “AtMDL2”: Top 300 genes co-expressed with *At*MDL2 according to ATTED-II (http://atted.jp/). Highlighted in red are those genes that overlap with genes that are co-expressed with *At*MDL1.Sheet”AtMDL3”: Top 300 genes co-expressed with *At*MDL3 according to ATTED-II (http://atted.jp/) Sheet “overrepresented BINs”: Results of overrepresentation analysis using PageMan (http://mapman.mpimp-golm.mpg.de/general/ora/ora.shtml). Shown are the results of BINs that exhibit a p-value of <1e-10. Additional file 5: Figure S3. Multiple sequence alignment of plant MDL proteins. Plant MDL amino acid sequences were aligned with ClustalW2 (http://www.ebi.ac.uk/Tools/msa/clustalw2/) using standard parameters. Subsequently, the alignment was shaded with BoxShade 3.21 (http://www.ch.embnet.org/software/BOX_form.html) using standard settings. A black shade indicates identical amino acids; a grey shade denotes similar amino acids. In the consensus line, asterisks specify invariant amino acids and dots conserved amino acids with similar biophysical properties at a given position. Additional file 6: Figure S4. Multiple sequence alignment of plant MDL proteins highlighting the distribution of cysteine residues. Plant MDL amino acid sequences were aligned with ClustalW2 (http://www.ebi.ac.uk/Tools/msa/clustalw2/) using standard parameters. Cysteine residues are highlighted by yellow background. Additional file 7: File S2. Newick annotation of phylogenetic trees shown in Figure 3. Additional file 8: Figure S5. Multiple sequence alignments of *At*MDL1-, *At*MDL2- and *At*MDL3-like proteins. Plant MDL amino acid sequences were aligned with ClustalW2 (http://www.ebi.ac.uk/Tools/msa/clustalw2/) using standard parameters. Subsequently, the alignment was shaded with BoxShade 3.21 (http://www.ch.embnet.org/software/BOX_form.html) using standard settings. A black shade indicates identical amino acids; a grey shade denotes similar amino acids. In the consensus line, asterisks specify invariant amino acids and dots conserved amino acids with similar biophysical properties at a given position. Additional file 9: File S1. MIF/MDL protein sequences used in this study.

## Abbreviations

DDT: D-dopachrome tautomerase
MDL: MIF/DDT-like gene/protein
MIF: Macrophage migration inhibitory factor
NES: Nuclear export signal
PTS1: Peroxisomal targeting sequence 1
TPOR: Thiol-protein oxidoreductase
