# Supplementary figures and images for: Phylogeny and evolution of plant macrophage migration inhibitory factor/D-dopachrome tautomerase-like proteins
Source: BMC Evol Biol. 2015 Apr 14;15:64. doi: 10.1186/s12862-015-0337-x (PMC4407349; doi:10.1186/s12862-015-0337-x)

## Supplemental Figure 1

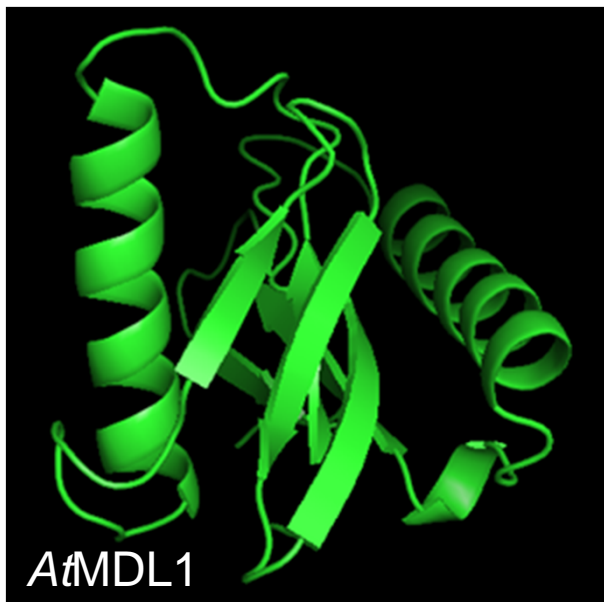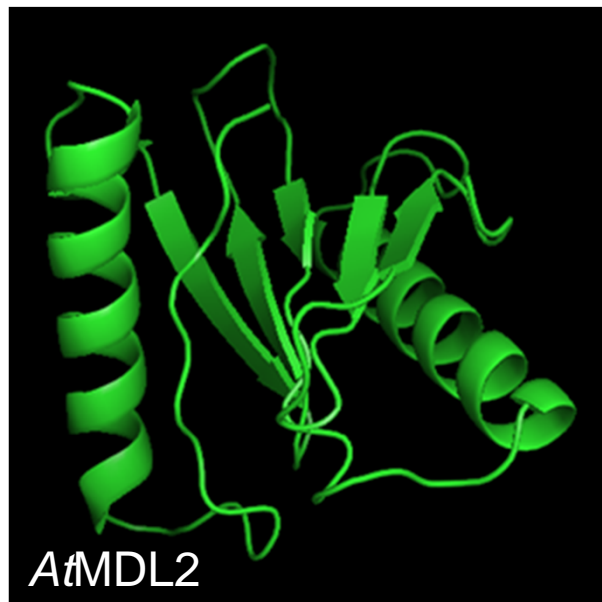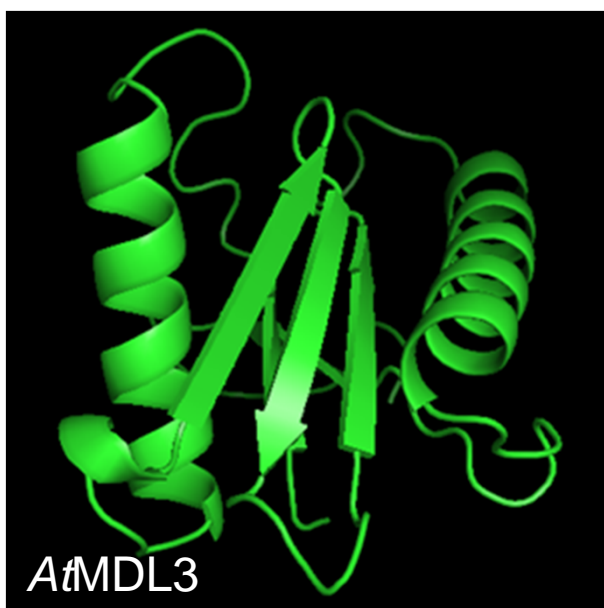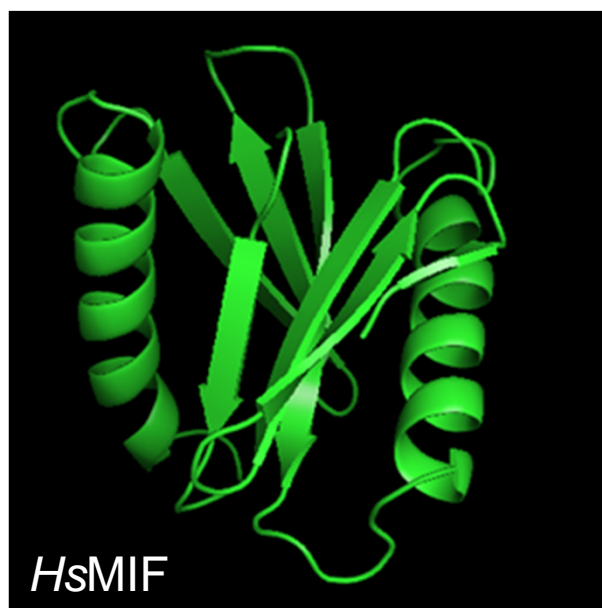

Supplement: Additional file 1: Figure S1. — Ab initio prediction of HsMIF/AtMDL 3D structures with QUARK. Amino acid sequences of the AtMDLs were subjected to analysis via the QUARK 3D structure prediction server (http://zhanglab.ccmb.med.umich.edu/QUARK/) and rendered with PyMOL (http://www.pymol.org/). The predicted 3D structures (ribbon models) of AtMDL1, AtMDL2 and AtMDL3 are visualized in comparison to the predicted structure of HsMIF. [file 12862_2015_337_MOESM1_ESM.pdf]
