# Supplementary material for: Phylogeny and evolution of plant macrophage migration inhibitory factor/D-dopachrome tautomerase-like proteins
Source: BMC Evol Biol. 2015 Apr 14;15:64. doi: 10.1186/s12862-015-0337-x (PMC4407349; doi:10.1186/s12862-015-0337-x)

# Supplemental Figure 2A

*AtMDL1*

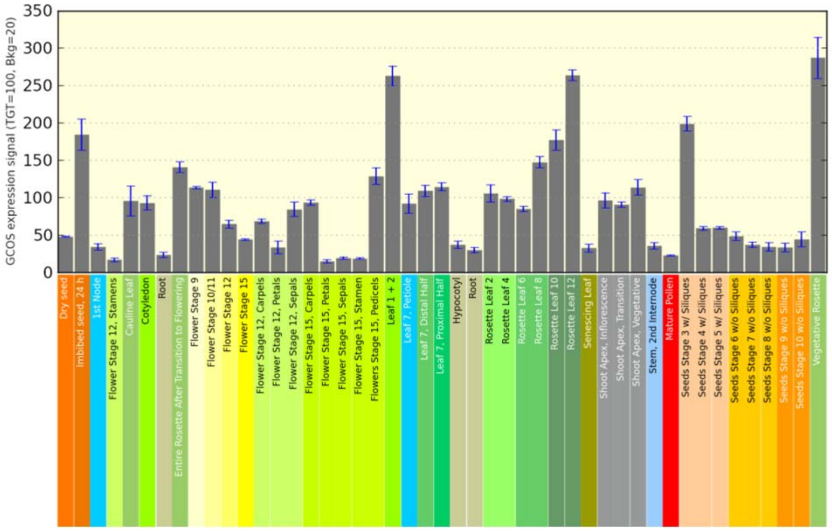


*AtMDL2*

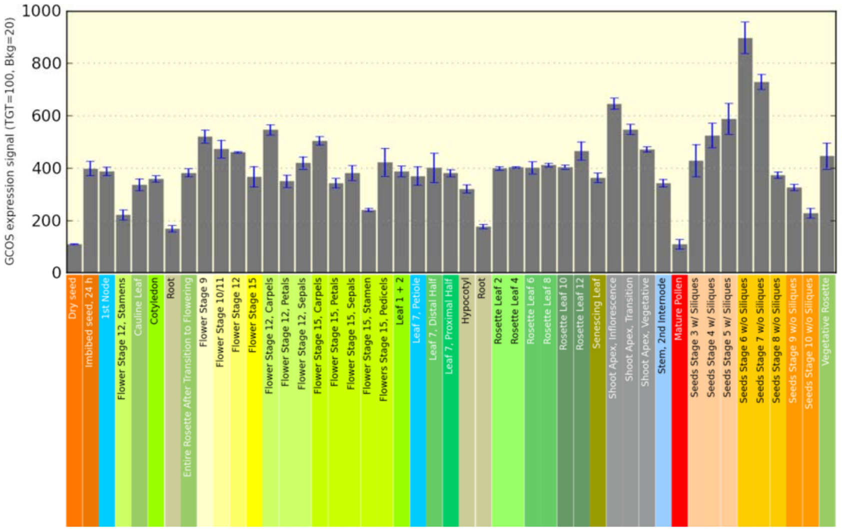


*AtMDL3*

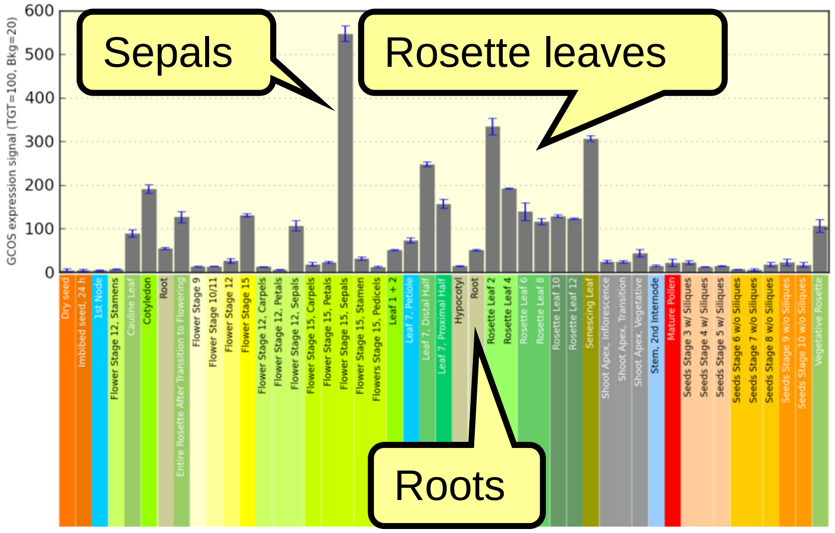

Supplemental Figure 2B

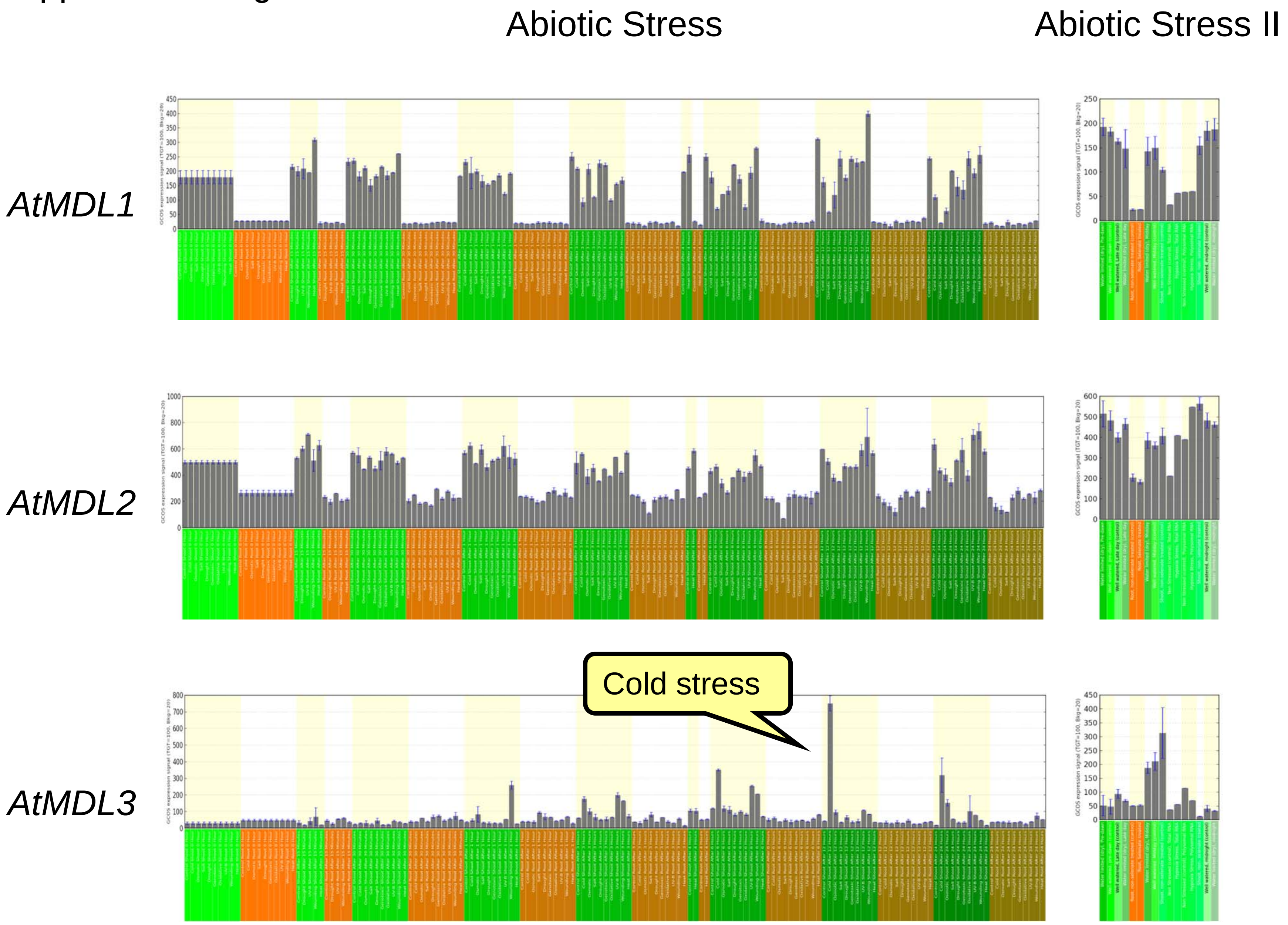

# Supplemental Figure 2C

Biotic Stress

Biotic Stress II

*AtMDL1*

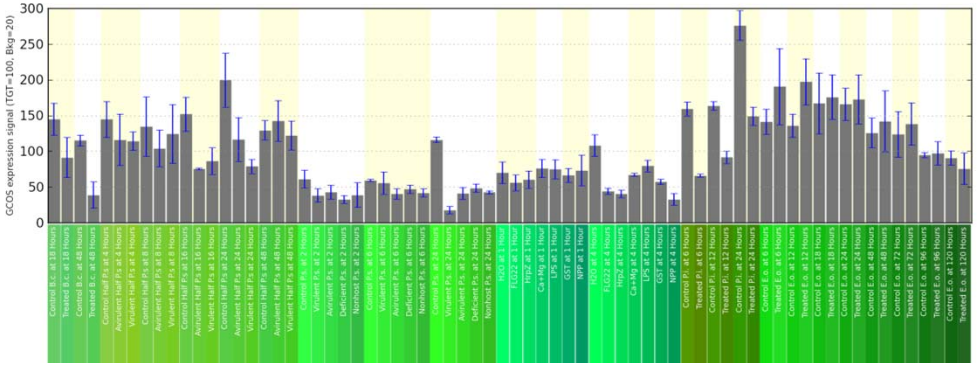

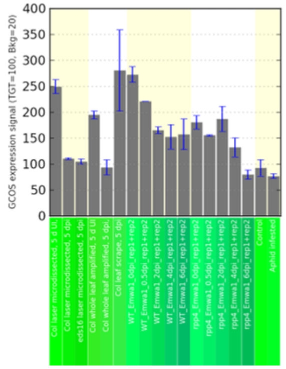

*AtMDL2*

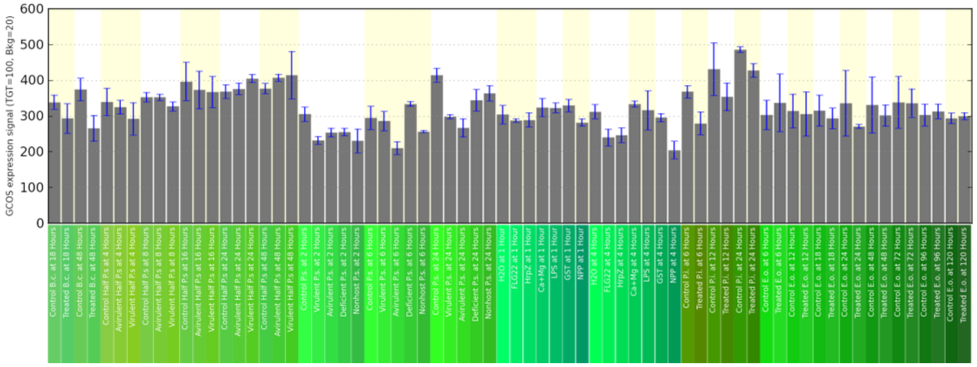

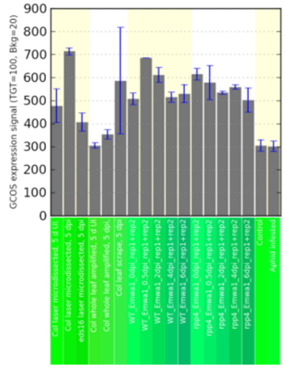

*AtMDL3*

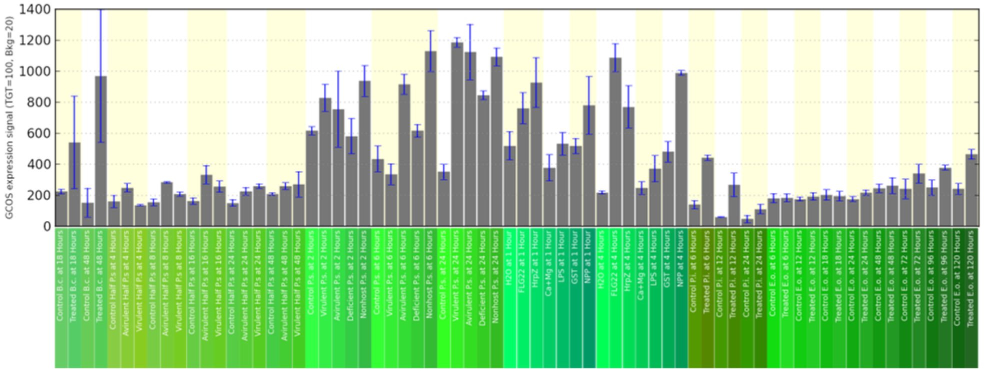

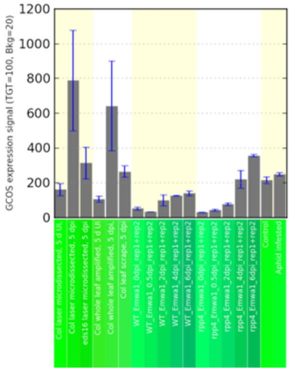

Supplement: Additional file 2: Figure S2. — AtMDL expression profiles. Gene expression data for AtMDL1, AtMDL2 and AtMDL3 are shown according to the Arabidopsis eFP browser and represent data sets from development (A), abiotic (B) and biotic (C) stress. [file 12862_2015_337_MOESM2_ESM.pdf]
