# Supplementary material for: Phylogeny and evolution of plant macrophage migration inhibitory factor/D-dopachrome tautomerase-like proteins
Source: BMC Evol Biol. 2015 Apr 14;15:64. doi: 10.1186/s12862-015-0337-x (PMC4407349; doi:10.1186/s12862-015-0337-x)
Supplement: Additional file 5: Figure S3. — Multiple sequence alignment of plant MDL proteins. Plant MDL amino acid sequences were aligned with ClustalW2 (http://www.ebi.ac.uk/Tools/msa/clustalw2/) using standard parameters. Subsequently, the alignment was shaded with BoxShade 3.21 (http://www.ch.embnet.org/software/BOX_form.html) using standard settings. A black shade indicates identical amino acids; a grey shade denotes similar amino acids. In the consensus line, asterisks specify invariant amino acids and dots conserved amino acids with similar biophysical properties at a given position. [file 12862_2015_337_MOESM5_ESM.pdf]

## Supplemental Figure 3. Multiple sequence alignment of plant MDL proteins.

```
H._vulgare_BAJ88384                1 MPQLSLSTNVP-VDAVVAADILRDCSRALARIIGKPESYVTVSIDGSVPTSFA-GSEE-PAAYGEIMSIGGLGPGVN----GKLSAALADILEAKLSISP
B._distachyon_XP_003577817         1 MPQLSISTNVP-VDAIVAADILKDCSRALAKIIGKPESYVMVSINGCVPTSFA-GSEE-PAAYGEIMSIGGLGPGVN----GKLSAALAEILETKLSISR
O._sativa_NP_001058472             1 MPQLSLTTNVP-VDAVVAADIIKDCSKALARIIGKPESYVMVSISGSVPMSFA-ASEE-PAAYGELMSIGGIGPGVN----GKLSAALAEILETKLSVSR
S._bicolor_XP_002438947            1 MPTLNLRTNVP-VDAVVAADILKDCSKAVARIIGKPESYVMVSINGSVPMSFA-GSEE-PAAYGELVSIGGIGPGVN----GKLSAAVAEVLETKLSVSR
Z._mays_NP_001150913               1 MPTLNLSTNVP-VDAVVAADILRDCSKAVARIIGKPESYVMVSVNGSVPMSFA-ASEE-PAAYGELVSIGGIGPGVN----GKLSAAVAEVLEAKLSVSR
S._lycopersicum_XP_004242799       1 MPTLNLFTNLP-VDAVIASDILKDATKAVAKIIGKPESYVMILLNGGVPIAFA-GTEA-PAAYGELISIGGLGPSVN----GKLSSTIAEILQTKLSIDS
A._thaliana_AtMDL1                 1 MPTLNLFTNIP-VDAVTCSDILKDATKAVAKIIGKPESYVMILLNSGVPIAFA-GTEE-PAAYGELISIGGLGPGVN----GKLSETISEILQIKLSIDS
P._persica_XP_007225890            1 MPTLNLFTNLP-VDAVVASDILKDATKAVSKIIGKPESYVMILLNGSVPMAFA-GTEE-PAAYGELISIGGIGPSVN----GKLSSTIAEILETKLSIDS
G._max_NP_001238163                1 MPTLDLFTNVP-VDTVVASDILRDATKAVAKIIGKPESYVMILLNGGVPIAFA-GTEE-PAAYGELISIGGLGPSVN----GKLSSTIAEILETKLYIDS
L._japonicus_AFK37854              1 MPTLNLFTNIP-VDAVVASDILRDATKAVAKIIGKPESYVMILLNGGVPIAFG-GTEE-PAAYGELISIGGLGPSVN----GKLSSTIAEILQTKLYIDG
V._vinifera_XP_002264373           1 MPTLNLFTNVP-VDAVVASDILKDCTKAVAKIIGKPESYVMILLNGGVPIEFA-GTEE-PAAYGELISIGGLGPSVN----GKLSSTIAEIIQTKLSIDS
P._sitchensis_ABK23267             1 MPTLNLSTNVP-VDSVVSSDILKDASKSVARIIGKPESYVMVLLKGGVPMLFG-GSEE-PAAYGEVVSIGGLGPGVN----GKLSAAIADILESKLSVDK
S._moellendorffii_XP_002961952     1 MPTLNLSTNVP-ADSIVSSDILKDASKAVSRIIGKPEQYVMIVLKDSVPMSFG-GTEE-PAAYGEVISIGGLGPSVN----KELSEALSEILESKLSVPP
C._reinhardtii_XP_001691775        1 MPTLNIITNVA-GDRVTTSDVLKALSKAVASSVGKPEQWVMASVTTDKPMIYG-GTEE-PCAFGYLMSIGSIGGDKN----KKISAAICEVLTAKLGVPA
V._carteri_XP_002955179            1 MPTLNVITNVP-CDRVTSSDVLKALSKAVSKSVGKPEQWVMCSLTTDKPMIYG-GSEE-PCAFGYFMSIGAIGGDVNQPNIRQISAAICEVLSTHLGVPA
P._patens_XP_001768921             1 MPTLNIQTNVP-LDGVVTSDILKDASKAVAQILSKPESYVLISLRGGIPMSFG-GTEE-PAAYGELISIGGVGPDTN----KRLSAAISDILKTKLSVPS
S._moellendorffii_XP_002966661     1 MPTLNISTNVP-LDGVSTSDILKDASRTVARVLGKPESYVMIIINGAVPISFG-GSEE-PAAYGELVSIGAISPDSN----KKLSKAIAELLQSKLAVPP
S._moellendorffii_XP_002964007     1 MPILTISTNTS-VDSSTSFYILQEATAAVARVLGKPESSMMVLLNDRVPILFG-GSRE-AAAYGELVSIGAIAPDSN----RKVSKALAGILESRLSVPP
S._moellendorffii_XP_002983015     1 MPVLTIHTNVVLLDGSLMSSVVSKLSHEVAKTTGKPESYVMVLLHGGVTLAFQ-GSHESAAAYGELVSIGGLSPGVN----QDLCKAIARVLEEELKVPP
S._lycopersicum_XP_004249062       1 MPCLNISTNVN-LEGVDTSSVLSEATSTVAKLIGKPEAYVMIVLKGSVPMAFG-GTEQ-PAAYGELVSIGGLNADVN----KKLSAAIADILETKLSIPK
A._thaliana_AtMDL2                 1 MPCLNLSTNVN-LDGVDTSSILSEASSTVAKIIGKPENYVMIVLKGSVPMSFG-GTED-PAAYGELVSIGGLNADVN----KKLSAAVSAILETKLSVPK
G._max_NP_001236304                1 MPCLNLSTNVS-LEGVDTSSILAEATSSVASIIGKPEAYVMIVLKGSVPIAFG-GNEQ-PAAYGELVSIGGLNPSVN----KELSAAIASILETKLSVPK
V._vinifera_XP_002263560           1 MPCLNLSTNVS-LDGVDTSSILSEATSTVAKIIGKPEAYVMIVLKGSVPIAFG-GTEQ-PAAYGELVSIGGLNPGTN----KELSAAISAILETKLSVPK
P._persica_XP_007207497            1 MPCLNISANVS-LEGVDTSSILSEATSTVAKIISKPEAYVMIVLKGSVPIAFG-GTEQ-PAAYGELVSIGGLNPDVN----KKLSAAIAAILETKLSVPK
G._max_NP_001237629                1 MPCLNLSTNVN-LDGIDTSSILSEATSTVASIIGKPEAYVMIVLKGSVPISHG-GSEQ-PAAYGELVSIGGLSPDVN----KKLSAGIASILENKLSVPK
L._japonicus_AFK37368              1 MPCLNLSTNVN-LDGVDTSSILSEATSTVATLIGKPEAYVMIVLKGSVPVSFG-GTEQ-PAAYGELVSIGGLNPDVN----KKLSAAIASILETKLSVPK
H._vulgare_BAJ92045                1 MPCLNVSTNVN-LEGVDTSAVLADASSTVATIIGKPEAYVMVVLKGSVPMAFG-GTQE-PAAYGELVSIGGLNPDVN----KKLSAGIASILESKLSISK
B._distachyon_XP_003579042         1 MPCLNVSTNVN-LDGVDTSAVLADASSAVATIIGKPEAYVMVVLKGSVPMAFG-GTQE-PAAYGELVSIGGLSPDVN----KKLSAGIAAILESKLSIPK
O._sativa_ABG22330                 1 MPCLNVSTNVN-LDGVDTSAVLADASKTVATIIGKPEAYVMVVLKGSVPMAFG-GTQE-PAAYGELVSIGGLNPDVN----KKLSAGIASILESKLSIPK
S._bicolor_XP_002441679            1 MPCLNVSTNVN-LEGVDTSVILAEASKSVANIIGKPEAYVMVVLKGSVPMAFG-GTQE-PAAYGELVSIGGLNPDVN----KKLSAGIASILESKLSVPK
Z._mays_XP_008677331               1 MPCLNVSTNVN-LEGVDTSAILAEASKSVANIIGKPEAYVMVVLKGSVPMAFG-GTQE-PAAYGELVSIGGLNPDVN----KKLSAGIASILESKLSVPK
P._sitchensis_ABK23881             1 MPSLNISTNVP-LEGLNTSEILSETSKSVAKIIGKPEAYVMVQLKGSVAISFG-GTEE-PAAYGELVSIGGLGSDTN----KKLSAAIATILDTKLKVPK
S._lycopersicum_XP_004249685       1 MPCFNLSTNVN-LDGVDTSDFFSEATKAVSSIIGKPENFVMVVLKGSVDISFG-GNKE-PAAFAEIVSMGGINSDVK----RKLIATLGGICQNRFSIPR
V._vinifera_XP_002264120           1 MPCVDISTNVN-LEGVDADPIFSDVTKAVASIIGKPENYVMVLLKGSVAISFE-GNKE-PAAFAEIISMGGINSEVK----RKLIATIGTILQTKLSIPR
G._max_ACU19241                    1 MPCLYITTNLN-LDGVDTNPVFSEATTAVSTIIGKPEKFVMVILKSSVPISFE-GNKE-PAAYAEIVSMGGISTEVK----RKLIATIGTILQSNLSIPR
L._japonicus_AFK35159              1 MPCLYIHTNIN-LDGVDTDSIFSEATTAVSTIIGKPEKFVMVLLKGSVPISFE-CNKE-PAAYAEIISMGGINSEVK----KKLIYTIGTILQSKLSIPR
A._thaliana_AtMDL3                 1 MPCLYITTNVN-FDGVNTDPFYSEVTKAVASIVGRPQNLVMVVLKGSVEIVFG-GNKE-AAAYAEIVSMGGITKQVK----RELIATVGSILHTHFSIHP
P._persica_XP_007207503            1 MPCLYISTNVN-LDGFDTDSIFSEATKAISSITGKPEDYVMVLLKGSVPISFGRSTSE-PAAYGELVAMGGINKPVK----RQLIATLGTIMEAKLSIPK
consensus                          1 ** .....*.  .... ..... ............*. ............ .. .... ..*.......*.. ....    ........ ..........

H._vulgare_BAJ88384               94 SRFYVKFDDVQGYNVGFNGTTF----------
B._distachyon_XP_003577817        94 SRFYVKFDDVQGYNLGFNGSTF----------
O._sativa_NP_001058472            94 SRFYVKFDDVKGFNLGFNGSTF----------
S._bicolor_XP_002438947           94 SRFYIKFDDVQRSNFGFNGSTF----------
Z._mays_NP_001150913              94 SRFYIKFDDVRGHNFGFNGSTF----------
S._lycopersicum_XP_004242799      94 DRFYIKFYDSPRPFFGFNGSTF----------
A._thaliana_AtMDL1                94 SRFYIKFYDSPRPFFGYNGSTF----------
P._persica_XP_007225890           94 SRFYIKFYDVERPFFGFNGSTF----------
G._max_NP_001238163               94 SRFYIKFYDVQRSFFGFNGSTF----------
L._japonicus_AFK37854             94 SRFYIKFYDVQRSFFGFNGSTF----------
V._vinifera_XP_002264373          94 ARFYVKFYDVERSFFGFNGSTF----------
P._sitchensis_ABK23267            94 SRFYIKFYDVEGSYFGFRGSTF----------
S._moellendorffii_XP_002961952    94 SRFYIKFYDVQRSFFGWNGSTF----------
C._reinhardtii_XP_001691775       94 NRVYIQFSDAKASDVGWDGSTFA---------
V._carteri_XP_002955179           98 ARVYIEFSDVNASDVGWNGSTFV---------
P._patens_XP_001768921            94 NRFYIKFFDVKRSDMGWNGSTF----------
S._moellendorffii_XP_002966661    94 NRFYIKFYDVKGSNFGWNGSTF----------
S._moellendorffii_XP_002964007    94 SRFYIKFYDVKGSNVGYNGSTY----------
S._moellendorffii_XP_002983015    96 SRCYIKFYQEQACVLRIWCSVSLFLFDEILSF
S._lycopersicum_XP_004249062      94 SRFFLKFYDTKGSFFGWNGSTF----------
A._thaliana_AtMDL2                94 SRFFLKFYDTKGSFFGWNGATL----------
G._max_NP_001236304               94 SRFFLKFYDTKGSNFGWNGSTF----------
V._vinifera_XP_002263560          94 SRFFLKFYDSKGSNFGWNGSTF----------
P._persica_XP_007207497           94 SRFFLKFYDTKGSNFGWNGSTF----------
G._max_NP_001237629               94 SRFYLKFYDTKGSNFGWNGSTF----------
L._japonicus_AFK37368             94 SRFFLKFYDTKGSNFGWNGSTF----------
H._vulgare_BAJ92045               94 SRFYLKFHDSKRSDFGWNGTTF----------
B._distachyon_XP_003579042        94 SRFYLKFHDSKRSDFGWNGTTF----------
O._sativa_ABG22330                94 GRFYLKFYDSKRSDFGWNGTTF----------
S._bicolor_XP_002441679           94 SRFYLKFYDSKRSDFGWNGSTF----------
Z._mays_XP_008677331              94 SRFYLKFYDSKRSDFGWNGSTF----------
P._sitchensis_ABK23881            94 SRFYIKFYDVKRSDFGWNGTTF----------
S._lycopersicum_XP_004249685      94 TRFFLKVYDTT---MATKFSKL----------
V._vinifera_XP_002264120          94 TRFFLKVYDTT---MAHKIAKL----------
G._max_ACU19241                   94 TRFFLKVFDVS---AFRTNSKM----------
L._japonicus_AFK35159             94 TRFFLKVFDTT---LFRNKSKL----------
A._thaliana_AtMDL3                94 TRFIFKVFDIN---SLPLPSKL----------
P._persica_XP_007207503           95 TRFFLKVVDIS---TATG-SKL----------
consensus                        101 .*......... . ........
```
