# Supplementary material for: Phylogeny and evolution of plant macrophage migration inhibitory factor/D-dopachrome tautomerase-like proteins
Source: BMC Evol Biol. 2015 Apr 14;15:64. doi: 10.1186/s12862-015-0337-x (PMC4407349; doi:10.1186/s12862-015-0337-x)
Supplement: Additional file 6: Figure S4. — Multiple sequence alignment of plant MDL proteins highlighting the distribution of cysteine residues. Plant MDL amino acid sequences were aligned with ClustalW2 (http://www.ebi.ac.uk/Tools/msa/clustalw2/) using standard parameters. Cysteine residues are highlighted by yellow background. [file 12862_2015_337_MOESM6_ESM.pdf]

Supplemental Figure 4. Multiple sequence alignment of plant MDL proteins highlighting the distribution of cysteine residues.

```
H._vulgare_BAJ88384                    MPQLSLSTNVP-VDAVVAADILRDCSRALARIIGKPESYVTVSIDGSVPT
B._distachyon_XP_003577817             MPQLSISTNVP-VDAIVAADILKDCSRALAKIIGKPESYVMVSINGCVPT
O._sativa_NP_001058472                 MPQLSLTTNVP-VDAVVAADIIKDCSKALARIIGKPESYVMVSISGSVPM
S._bicolor_XP_002438947                MPTLNLRTNVP-VDAVVAADILKDCSKAVARIIGKPESYVMVSINGSVPM
Z._mays_NP_001150913                   MPTLNLSTNVP-VDAVVAADILRDCSKAVARIIGKPESYVMVSVNGSVPM
S._lycopersicum_XP_004242799           MPTLNLFTNLP-VDAVIASDILKDATKAVAKIIGKPESYVMILLNGGVPI
A._thaliana_AtMDL1                     MPTLNLFTNIP-VDAVTCSDILKDATKAVAKIIGKPESYVMILLNSGVPI
P._persica_XP_007225890                MPTLNLFTNLP-VDAVVASDILKDATKAVSKIIGKPESYVMILLNGSVPM
G._max_NP_001238163                    MPTLDLFTNVP-VDTVVASDILRDATKAVAKIIGKPESYVMILLNGGVPI
L._japonicus_AFK37854                  MPTLNLFTNIP-VDAVVASDILRDATKAVAKIIGKPESYVMILLNGGVPI
V._vinifera_XP_002264373               MPTLNLFTNVP-VDAVVASDILKDCTKAVAKIIGKPESYVMILLNGGVPI
P._sitchensis_ABK23267                 MPTLNLSTNVP-VDSVVSSDILKDASKSVARIIGKPESYVMVLLKGGVPM
S._moellendorffii_XP_002961952         MPTLNLSTNVP-ADSIVSSDILKDASKAVSRIIGKPEQYVMIVLKDSVPM
C._reinhardtii_XP_001691775            MPTLNIITNVA-GDRVTTSDVLKALSKAVASSVGKPEQWVMASVTTDKPM
V._carteri_XP_002955179                MPTLNVITNVP-CDRVTSSDVLKALSKAVSKSVGKPEQWVMCSLTTDKPM
P._patens_XP_001768921                 MPTLNIQTNVP-LDGVVTSDILKDASKAVAQILSKPESYVLISLRGGIPM
S._moellendorffii_XP_002966661         MPTLNISTNVP-LDGVSTSDILKDASRTVARVLGKPESYVMIIINGAVPI
S._moellendorffii_XP_002964007         MPILTISTNTS-VDSSTSFYILQEATAAVARVLGKPESSMMVLLNDRVPI
S._moellendorffii_XP_002983015         MPVLTIHTNVVLLDGSLMSSVVSKLSHEVAKTTGKPESYVMVLLHGGVTL
S._lycopersicum_XP_004249062           MPCLNISTNVN-LEGVDTSSVLSEATSTVAKLIGKPEAYVMIVLKGSVPM
A._thaliana_AtMDL2                     MPCLNLSTNVN-LDGVDTSSILSEASSTVAKIIGKPENYVMIVLKGSVPM
G._max_NP_001236304                    MPCLNLSTNVS-LEGVDTSSILAEATSSVASIIGKPEAYVMIVLKGSVPI
V._vinifera_XP_002263560               MPCLNLSTNVS-LDGVDTSSILSEATSTVAKIIGKPEAYVMIVLKGSVPI
P._persica_XP_007207497                MPCLNISANVS-LEGVDTSSILSEATSTVAKIISKPEAYVMIVLKGSVPI
G._max_NP_001237629                    MPCLNLSTNVN-LDGIDTSSILSEATSTVASIIGKPEAYVMIVLKGSVPI
L._japonicus_AFK37368                  MPCLNLSTNVN-LDGVDTSSILSEATSTVATLIGKPEAYVMIVLKGSVPV
H._vulgare_BAJ92045                    MPCLNVSTNVN-LEGVDTSAVLADASSTVATIIGKPEAYVMVVLKGSVPM
B._distachyon_XP_003579042             MPCLNVSTNVN-LDGVDTSAVLADASSAVATIIGKPEAYVMVVLKGSVPM
O._sativa_ABG22330                     MPCLNVSTNVN-LDGVDTSAVLADASKTVATIIGKPEAYVMVVLKGSVPM
S._bicolor_XP_002441679                MPCLNVSTNVN-LEGVDTSVILAEASKSVANIIGKPEAYVMVVLKGSVPM
Z._mays_XP_008677331                   MPCLNVSTNVN-LEGVDTSAILAEASKSVANIIGKPEAYVMVVLKGSVPM
P._sitchensis_ABK23881                 MPSLNISTNVP-LEGLNTSEILSETSKSVAKIIGKPEAYVMVQLKGSVAI
S._lycopersicum_XP_004249685           MPCFNLSTNVN-LDGVDTSDFFSEATKAVSSIIGKPENFVMVVLKGSVDI
V._vinifera_XP_002264120               MPCVDISTNVN-LEGVDADPIFSDVTKAVASIIGKPENYVMVLLKGSVAI
G._max_ACU19241                        MPCLYITTNLN-LDGVDTNPVFSEATTAVSTIIGKPEKFVMVILKSSVPI
L._japonicus_AFK35159                  MPCLYIHTNIN-LDGVDTDSIFSEATTAVSTIIGKPEKFVMVLLKGSVPI
A._thaliana_AtMDL3                     MPCLYITTNVN-FDGVNTDPFYSEVTKAVASIVGRPQNLVMVVLKGSVEI
P._persica_XP_007207503                MPCLYISTNVN-LDGFDTDSIFSEATKAISSITGKPEDYVMVLLKGSVPI
                                       ** . : :*    :      .    :  ::   .:*:  :   :
```

```
H._vulgare_BAJ88384                 SFA-GSEE-PAAYGEIMSIGGLGPGVN----GKLSAALADILEAKLSISP
B._distachyon_XP_003577817          SFA-GSEE-PAAYGEIMSIGGLGPGVN----GKLSAALAEILETKLSISR
O._sativa_NP_001058472              SFA-ASEE-PAAYGELMSIGGIGPGVN----GKLSAALAEILETKLSVSR
S._bicolor_XP_002438947             SFA-GSEE-PAAYGELVSIGGIGPGVN----GKLSAAVAEVLETKLSVSR
Z._mays_NP_001150913                SFA-ASEE-PAAYGELVSIGGIGPGVN----GKLSAAVAEVLEAKLSVSR
S._lycopersicum_XP_004242799        AFA-GTEA-PAAYGELISIGGLGPSVN----GKLSSTIAEILQTKLSIDS
A._thaliana_AtMDL1                  AFA-GTEE-PAAYGELISIGGLGPGVN----GKLSETISEILQIKLSIDS
P._persica_XP_007225890             AFA-GTEE-PAAYGELISIGGIGPSVN----GKLSSTIAEILETKLSIDS
G._max_NP_001238163                 AFA-GTEE-PAAYGELISIGGLGPSVN----GKLSSTIAEILETKLYIDS
L._japonicus_AFK37854               AFG-GTEE-PAAYGELISIGGLGPSVN----GKLSSTIAEILQTKLYIDG
V._vinifera_XP_002264373            EFA-GTEE-PAAYGELISIGGLGPSVN----GKLSSTIAEIIQTKLSIDS
P._sitchensis_ABK23267              LFG-GSEE-PAAYGEVVSIGGLGPGVN----GKLSAAIADILESKLSVDK
S._moellendorffii_XP_002961952      SFG-GTEE-PAAYGEVISIGGLGPSVN----KELSEALSEILESKLSVPP
C._reinhardtii_XP_001691775         IYG-GTEE-PCAFGYLMSIGSIGGDKN----KKISAAICEVLTAKLGVPA
V._carteri_XP_002955179             IYG-GSEE-PCAFGYFMSIGAIGGDVNQPNIRQISAAICEVLSTHLGVPA
P._patens_XP_001768921              SFG-GTEE-PAAYGELISIGGVGPDTN----KRLSAAISDILKTKLSVPS
S._moellendorffii_XP_002966661      SFG-GSEE-PAAYGELVSIGAISPDSN----KKLSKAIAELLQSKLAVPP
S._moellendorffii_XP_002964007      LFG-GSRE-AAAYGELVSIGAIAPDSN----RKVSKALAGILESRLSVPP
S._moellendorffii_XP_002983015      AFQ-GSHESAAAYGELVSIGGLSPGVN----QDLCKAIARVLEEELKVPP
S._lycopersicum_XP_004249062        AFG-GTEQ-PAAYGELVSIGGLNADVN----KKLSAAIADILETKLSIPK
A._thaliana_AtMDL2                  SFG-GTED-PAAYGELVSIGGLNADVN----KKLSAAVSAILETKLSVPK
G._max_NP_001236304                 AFG-GNEQ-PAAYGELVSIGGLNPSVN----KELSAAIASILETKLSVPK
V._vinifera_XP_002263560            AFG-GTEQ-PAAYGELVSIGGLNPGTN----KELSAAISAILETKLSVPK
P._persica_XP_007207497             AFG-GTEQ-PAAYGELVSIGGLNPDVN----KKLSAAIAAILETKLSVPK
G._max_NP_001237629                 SHG-GSEQ-PAAYGELVSIGGLSPDVN----KKLSAGIASILENKLSVPK
L._japonicus_AFK37368               SFG-GTEQ-PAAYGELVSIGGLNPDVN----KKLSAAIASILETKLSVPK
H._vulgare_BAJ92045                 AFG-GTQE-PAAYGELVSIGGLNPDVN----KKLSAGIASILESKLSISK
B._distachyon_XP_003579042          AFG-GTQE-PAAYGELVSIGGLSPDVN----KKLSAGIAAILESKLSIPK
O._sativa_ABG22330                  AFG-GTQE-PAAYGELVSIGGLNPDVN----KKLSAGIASILESKLSIPK
S._bicolor_XP_002441679             AFG-GTQE-PAAYGELVSIGGLNPDVN----KKLSAGIASILESKLSVPK
Z._mays_XP_008677331                AFG-GTQE-PAAYGELVSIGGLNPDVN----KKLSAGIASILESKLSVPK
P._sitchensis_ABK23881              SFG-GTEE-PAAYGELVSIGGLGSDTN----KKLSAAIATILDTKLKVPK
S._lycopersicum_XP_004249685        SFG-GNKE-PAAFAEIVSMGGINSDVK----RKLIATLGGICQNRFSIPR
V._vinifera_XP_002264120            SFE-GNKE-PAAFAEIISMGGINSEVK----RKLIATIGTILQTKLSIPR
G._max_ACU19241                     SFE-GNKE-PAAYAEIVSMGGISTEVK----RKLIATIGTILQSNLSIPR
L._japonicus_AFK35159               SFE-CNKE-PAAYAEIISMGGINSEVK----KKLIYTIGTILQSKLSIPR
A._thaliana_AtMDL3                  VFG-GNKE-AAAYAEIVSMGGITKQVK----RELIATVGSILHTHFSIHP
P._persica_XP_007207503             SFGRSTSE-PAAYGELVAMGGINKPVK----RQLIATLGTIMEAKLSIPK
                                     .   .   ..*:. .:::*.:    :      :   :  :   .: :
```

```
H._vulgare_BAJ88384                 SRFYVKFDDVQGYNVGFNGTTF----------
B._distachyon_XP_003577817          SRFYVKFDDVQGYNLGFNGSTF----------
O._sativa_NP_001058472              SRFYVKFDDVKGFNLGFNGSTF----------
S._bicolor_XP_002438947             SRFYIKFDDVQRSNFGFNGSTF----------
Z._mays_NP_001150913                SRFYIKFDDVRGHNFGFNGSTF----------
S._lycopersicum_XP_004242799        DRFYIKFYDSPRPFFGFNGSTF----------
A._thaliana_AtMDL1                  SRFYIKFYDSPRPFFGYNGSTF----------
P._persica_XP_007225890             SRFYIKFYDVERPFFGFNGSTF----------
G._max_NP_001238163                 SRFYIKFYDVQRSFFGFNGSTF----------
L._japonicus_AFK37854               SRFYIKFYDVQRSFFGFNGSTF----------
V._vinifera_XP_002264373            ARFYVKFYDVERSFFGFNGSTF----------
P._sitchensis_ABK23267              SRFYIKFYDVEGSYFGFRGSTF----------
S._moellendorffii_XP_002961952      SRFYIKFYDVQRSFFGWNGSTF----------
C._reinhardtii_XP_001691775         NRVYIQFSDAKASDVGWDGSTFA---------
V._carteri_XP_002955179             ARVYIEFSDVNASDVGWNGSTFV---------
P._patens_XP_001768921              NRFYIKFFDVKRSDMGWNGSTF----------
S._moellendorffii_XP_002966661      NRFYIKFYDVKGSNFGWNGSTF----------
S._moellendorffii_XP_002964007      SRFYIKFYDVKGSNVGYNGSTY----------
S._moellendorffii_XP_002983015      SRCYIKFYQEQACVLRIWCSVSLFLFDEILSF
S._lycopersicum_XP_004249062        SRFFLKFYDTKGSFFGWNGSTF----------
A._thaliana_AtMDL2                  SRFFLKFYDTKGSFFGWNGATL----------
G._max_NP_001236304                 SRFFLKFYDTKGSNFGWNGSTF----------
V._vinifera_XP_002263560            SRFFLKFYDSKGSNFGWNGSTF----------
P._persica_XP_007207497             SRFFLKFYDTKGSNFGWNGSTF----------
G._max_NP_001237629                 SRFYLKFYDTKGSNFGWNGSTF----------
L._japonicus_AFK37368               SRFFLKFYDTKGSNFGWNGSTF----------
H._vulgare_BAJ92045                 SRFYLKFHDSKRSDFGWNGTTF----------
B._distachyon_XP_003579042          SRFYLKFHDSKRSDFGWNGTTF----------
O._sativa_ABG22330                  GRFYLKFYDSKRSDFGWNGTTF----------
S._bicolor_XP_002441679             SRFYLKFYDSKRSDFGWNGSTF----------
Z._mays_XP_008677331                SRFYLKFYDSKRSDFGWNGSTF----------
P._sitchensis_ABK23881              SRFYIKFYDVKRSDFGWNGTTF----------
S._lycopersicum_XP_004249685        TRFFLKVYDTT---MATKFSKL----------
V._vinifera_XP_002264120            TRFFLKVYDTT---MAHKIAKL----------
G._max_ACU19241                     TRFFLKVFDVS---AFRTNSKM----------
L._japonicus_AFK35159               TRFFLKVFDTT---LFRNKSKL----------
A._thaliana_AtMDL3                  TRFIFKVFDIN---SLPLPSKL----------
P._persica_XP_007207503             TRFFLKVVDIS---TATG-SKL----------
                                     *  .:. :          :
```
