# Supplementary material for: Phylogeny and evolution of plant macrophage migration inhibitory factor/D-dopachrome tautomerase-like proteins
Source: BMC Evol Biol. 2015 Apr 14;15:64. doi: 10.1186/s12862-015-0337-x (PMC4407349; doi:10.1186/s12862-015-0337-x)
Supplement: Additional file 7: File S2. — Newick annotation of phylogenetic trees shown in Figure 3. [file 12862_2015_337_MOESM7_ESM.pdf]

Supplemental file 2. Newick annotation of phylogenetic trees shown in Figure 3.

Neighbor-Joining Tree

((((((((((G._max_NP_001236304:0.04588624,V._vinifera_XP_002263560:0.04107028)0.3600:0.00482061,P._persica_XP_007207497:0.03430982)0.4900:0.01400451,(G._max_NP_001237629:0.06476033,L._japonicus_AFK37368:0.02219619)0.4900:0.01208245)0.2700:0.00681532,(A._thaliana_AtMDL2:0.07174780,S._lycopersicum_XP_004249062:0.04999134)0.4200:0.00568468)0.9800:0.03987451,((S._bicolor_XP_002441679:0.00457666,Z._mays_XP_008677331:0.00411899)1.0000:0.02550626,(O._sativa_ABG22330:0.01995561,(B._distachyon_XP_003579042:0.01897562,H._vulgare_BAJ92045:0.02450264)0.5200:0.00613134)0.9500:0.02884156)0.9900:0.05474699)0.7900:0.01857978,P._sitchensis_ABK23881:0.13783373)0.5900:0.01727805,(P._persica_XP_007207503:0.15870416,(A._thaliana_AtMDL3:0.22807663,((G._max_ACU19241:0.09887462,L._japonicus_AFK35159:0.07969681)0.9500:0.05542273,(S._lycopersicum_XP_004249685:0.11810633,V._vinifera_XP_002264120:0.11403653)0.7200:0.02270227)0.5400:0.01634301)0.5700:0.01308034)1.0000:0.14470752)0.5500:0.03508263,(P._patens_XP_001768921:0.15834646,(S._moellendorffii_XP_002961952:0.12062722,(P._sitchensis_ABK23267:0.09823158,((((B._distachyon_XP_003577817:0.04575734,H._vulgare_BAJ88384:0.05859049)0.9300:0.02567643,O._sativa_NP_001058472:0.06562792)0.8800:0.03223029,(S._bicolor_XP_002438947:0.02614541,Z._mays_NP_001150913:0.04341980)0.8900:0.02429145)1.0000:0.07616627,(((A._thaliana_AtMDL1:0.05207125,S._lycopersicum_XP_004242799:0.04358092)0.4500:0.01730107,P._persica_XP_007225890:0.04791633)0.1900:0.00070512,(V._vinifera_XP_002264373:0.05657902,(G._max_NP_001238163:0.02653222,L._japonicus_AFK37854:0.02564170)0.7100:0.01298620)0.2200:0.00636009)1.0000:0.08157829)0.3600:0.00808635)0.5800:0.01605757)0.7400:0.01828398)0.4800:0.01768331)0.3500:0.02132685,(S._moellendorffii_XP_002966661:0.11962405,S._moellendorffii_XP_002964007:0.21950639)0.5400:0.02647439)0.3700:0.01767299,S._moellendorffii_XP_002983015:0.30259454,((C._reinhardtii_XP_001691775:0.09573710,V._carteri_XP_002955179:0.10253876)1.0000:0.13417537,(H._sapiens_HsMIF:0.29386760,H._sapiens_HsDDT:0.35830631)0.9900:0.08604971)0.9200:0.06789560);

Maximum Likelihood Tree

(((((((((L._japonicus_AFK37854:0.02560130,G._max_NP_001238163:0.02650131)0.5900:0.01875560,V._vinifera_XP_002264373:0.05207026)0.1100:0.00000008,(S._lycopersicum_XP_004242799:0.02758863,A._thaliana_AtMDL1:0.07803840)0.7700:0.03699779)0.5200:0.02883939,P._persica_XP_007225890:0.03919830)0.9900:0.10037597,(S._bicolor_XP_002438947:0.00888205,(Z._mays_NP_001150913:0.04519429,(O._sativa_NP_001058472:0.04807890,(H._vulgare_BAJ88384:0.07085274,B._distachyon_XP_003577817:0.03416919)0.9100:0.04917604)0.9500:0.07750525)0.4900:0.01691654)0.9400:0.10833716)0.4000:0.04322303,P._sitchensis_ABK23267:0.10287490)0.6300:0.04788919,S._moellendorffii_XP_002961952:0.12951367)0.6200:0.07050599,(P._sitchensis_ABK23881:0.13422508,((G._max_NP_001237629:0.06107012,(L._japonicus_AFK37368:0.01875088,(((V._vinifera_XP_002263560:0.03363520,G._max_NP_001236304:0.05769313)0.4900:0.01466309,P._persica_XP_007207497:0.03128023)0.4300:0.01630440,(S._lycopersicum_XP_004249062:0.05486048,(A._thaliana_AtMDL2:0.03388689,(P._persica_XP_007207503:0.19247579,((S._lycopersicum_XP_004249685:0.12618367,A._thaliana_AtMDL3:0.34128159)0.5400:0.05538908,(V._vinifera_XP_002264120:0.12359939,(L._japonicus_AFK35159:0.06639070,G._max_ACU19241:0.13355475)0.8400:0.08900430)0.2800:0.02107051)0.7500:0.06189165)1.0000:0.35603040)0.4500:0.03919049)0.1800:0.02582256)0.1300:0.02165474)0.2500:0.01158738)0.5900:0.08001434,((S._bicolor_XP_002441679:0.00852588,Z._mays_XP_008677331:0.00000000)0.8400:0.02241252,(O._sativa_ABG22330:0.00876297,(B._distachyon_XP_003579042:0.02580221,H._vulgare_BAJ92045:0.01672237)0.7100:0.01748744)0.9100:0.03836428)0.9100:0.05970613)0.9300:0.09357738)0.7700:0.08478387)0.2400:0.03134427,(S._moellendorffii_XP_002966661:0.07862242,(S._moellendorffii_XP_002964007:0.25056608,S._moellendorffii_XP_002983015:0.67715473)0.4200:0.09244104)0.4700:0.13688579)0.6100:0.06369558,P._patens_XP_001768921:0.10514956,((C._reinhardtii_XP_001691775:0.07064352,V._carteri_XP_002955179:0.15260202)0.9100:0.18354398,(H._sapiens_HsMIF:0.43770245,H._sapiens_HsDDT:0.95656333)0.9600:0.45958451)0.9100:0.27599725);
