# Supplementary material for: Phylogeny and evolution of plant macrophage migration inhibitory factor/D-dopachrome tautomerase-like proteins
Source: BMC Evol Biol. 2015 Apr 14;15:64. doi: 10.1186/s12862-015-0337-x (PMC4407349; doi:10.1186/s12862-015-0337-x)
Supplement: Additional file 8: Figure S5. — Multiple sequence alignments of AtMDL1-, AtMDL2- and AtMDL3-like proteins. Plant MDL amino acid sequences were aligned with ClustalW2 (http://www.ebi.ac.uk/Tools/msa/clustalw2/) using standard parameters. Subsequently, the alignment was shaded with BoxShade 3.21 (http://www.ch.embnet.org/software/BOX_form.html) using standard settings. A black shade indicates identical amino acids; a grey shade denotes similar amino acids. In the consensus line, asterisks specify invariant amino acids and dots conserved amino acids with similar biophysical properties at a given position. [file 12862_2015_337_MOESM8_ESM.pdf]

Supplemental Figure 5. Multiple sequence alignments of *At*MDL1-, *At*MDL2- and *At*MDL3-like proteins.

Angiosperm MIF proteins with similarity to *AtMDL*1

```
S._lycopersicum_XP004242799    1 MPTLNLFTNLPVDAVIASDILKDATKAVAKIIGKPESYVMILLNGGVPIAFAGTEAPAAY
A._thaliana_AtMDL1             1 MPTLNLFTNIPVDAVTCSDILKDATKAVAKIIGKPESYVMILLNSGVPIAFAGTEEPAAY
G._max_NP001238163             1 MPTLDLFTNVPVDTVVASDILRDATKAVAKIIGKPESYVMILLNGGVPIAFAGTEEPAAY
L._japonicus_AFK37854          1 MPTLNLFTNIPVDAVVASDILRDATKAVAKIIGKPESYVMILLNGGVPIAFGGTEEPAAY
V._vinifera_XP002264373        1 MPTLNLFTNVPVDAVVASDILKDCTKAVAKIIGKPESYVMILLNGGVPIEFAGTEEPAAY
P._persica_XP007225890         1 MPTLNLFTNLPVDAVVASDILKDATKAVSKIIGKPESYVMILLNGSVPMAFAGTEEPAAY
B._distachyon_XP003577817      1 MPQLSISTNVPVDAIVAADILKDCSRALAKIIGKPESYVMVSINGCVPTSFAGSEEPAAY
H._vulgare_BAJ88384            1 MPQLSLSTNVPVDAVVAADILRDCSRALARIIGKPESYVTVSIDGSVPTSFAGSEEPAAY
O._sativa_NP001058472          1 MPQLSLTTNVPVDAVVAADIIKDCSKALARIIGKPESYVMVSISGSVPMSFAASEEPAAY
S._bicolor_XP002438947         1 MPTLNLRTNVPVDAVVAADILKDCSKAVARIIGKPESYVMVSINGSVPMSFAGSEEPAAY
Z._mays_NP001150913            1 MPTLNLSTNVPVDAVVAADILRDCSKAVARIIGKPESYVMVSVNGSVPMSFAASEEPAAY
consensus                      1 **.*...**.***.....**..*...*...*********...... **. *...*.****

S._lycopersicum_XP004242799   61 GELISIGGLGPSVNGKLSSTIAEILQTKLSIDSDRFYIKFYDSPRPFFGFNGSTF
A._thaliana_AtMDL1            61 GELISIGGLGPGVNGKLSETISEILQIKLSIDSSRFYIKFYDSPRPFFGYNGSTF
G._max_NP001238163            61 GELISIGGLGPSVNGKLSSTIAEILETKLYIDSSRFYIKFYDVQRSFFGFNGSTF
L._japonicus_AFK37854         61 GELISIGGLGPSVNGKLSSTIAEILQTKLYIDGSRFYIKFYDVQRSFFGFNGSTF
V._vinifera_XP002264373       61 GELISIGGLGPSVNGKLSSTIAEIIQTKLSIDSARFYVKFYDVERSFFGFNGSTF
P._persica_XP007225890        61 GELISIGGIGPSVNGKLSSTIAEILETKLSIDSSRFYIKFYDVERPFFGFNGSTF
B._distachyon_XP003577817     61 GEIMSIGGLGPGVNGKLSAALAEILETKLSISRSRFYVKFDDVQGYNLGFNGSTF
H._vulgare_BAJ88384           61 GEIMSIGGLGPGVNGKLSAALADILEAKLSISPSRFYVKFDDVQGYNVGFNGTTF
O._sativa_NP001058472         61 GELMSIGGIGPGVNGKLSAALAEILETKLSVSRSRFYVKFDDVKGFNLGFNGSTF
S._bicolor_XP002438947        61 GELVSIGGIGPGVNGKLSAAVAEVLETKLSVSRSRFYIKFDDVQRSNFGFNGSTF
Z._mays_NP001150913           61 GELVSIGGIGPGVNGKLSAAVAEVLEAKLSVSRSRFYIKFDDVRGHNFGFNGSTF
consensus                     61 **..****.**.****** ........**... .***.**.*. . ..*.**.**
```

Angiosperm MIF proteins with similarity to *AtMDL*2

```
S._lycopersicum_XP004249062    1 MPCLNISTNVNLEGVDTSSVLSEATSTVAKLIGKPEAYVMIVLKGSVPMAFGGTEQPAAY
A._thaliana_AtMDL2             1 MPCLNLSTNVNLDGVDTSSILSEASSTVAKIIGKPENYVMIVLKGSVPMSFGGTEDPAAY
G._max_NP001236304             1 MPCLNLSTNVSLEGVDTSSILAEATSSVASIIGKPEAYVMIVLKGSVPIAFGGNEQPAAY
V._vinifera_XP002263560        1 MPCLNLSTNVSLDGVDTSSILSEATSTVAKIIGKPEAYVMIVLKGSVPIAFGGTEQPAAY
P._persica_XP007207497         1 MPCLNISANVSLEGVDTSSILSEATSTVAKIISKPEAYVMIVLKGSVPIAFGGTEQPAAY
L._japonicus_AFK37368          1 MPCLNLSTNVNLDGVDTSSILSEATSTVATLIGKPEAYVMIVLKGSVPVSFGGTEQPAAY
G._max_NP001237629             1 MPCLNLSTNVNLDGIDTSSILSEATSTVASIIGKPEAYVMIVLKGSVPISHGGSEQPAAY
H._vulgare_BAJ92045            1 MPCLNVSTNVNLEGVDTSAVLADASSTVATIIGKPEAYVMVVLKGSVPMAFGGTQEPAAY
B._distachyon_XP003579042      1 MPCLNVSTNVNLDGVDTSAVLADASSAVATIIGKPEAYVMVVLKGSVPMAFGGTQEPAAY
O._sativa_ABG22330             1 MPCLNVSTNVNLDGVDTSAVLADASKTVATIIGKPEAYVMVVLKGSVPMAFGGTQEPAAY
S._bicolor_XP002441679         1 MPCLNVSTNVNLEGVDTSVILAEASKSVANIIGKPEAYVMVVLKGSVPMAFGGTQEPAAY
Z._mays_XP008677331            1 MPCLNVSTNVNLEGVDTSAILAEASKSVANIIGKPEAYVMVVLKGSVPMAFGGTQEPAAY
consensus                      1 *****.*.**.*.*.***..* .*...**..*.***.***.*******...**...****

S._lycopersicum_XP004249062   61 GELVSIGGLNADVNKKLSAAIADILETKLSIPKSRFFLKFYDTKGSFFGWNGSTF
A._thaliana_AtMDL2            61 GELVSIGGLNADVNKKLSAAVSAILETKLSVPKSRFFLKFYDTKGSFFGWNGATL
G._max_NP001236304            61 GELVSIGGLNPSVNKELSAAIASILETKLSVPKSRFFLKFYDTKGSNFGWNGSTF
V._vinifera_XP002263560       61 GELVSIGGLNPGTNKELSAAISAILETKLSVPKSRFFLKFYDSKGSNFGWNGSTF
P._persica_XP007207497        61 GELVSIGGLNPDVNKKLSAAIAAILETKLSVPKSRFFLKFYDTKGSNFGWNGSTF
L._japonicus_AFK37368         61 GELVSIGGLNPDVNKKLSAAIASILETKLSVPKSRFFLKFYDTKGSNFGWNGSTF
G._max_NP001237629            61 GELVSIGGLSPDVNKKLSAGIASILENKLSVPKSRFYLKFYDTKGSNFGWNGSTF
H._vulgare_BAJ92045           61 GELVSIGGLNPDVNKKLSAGIASILESKLSISKSRFYLKFHDSKRSDFGWNGTTF
B._distachyon_XP003579042     61 GELVSIGGLSPDVNKKLSAGIAAILESKLSIPKSRFYLKFHDSKRSDFGWNGTTF
O._sativa_ABG22330            61 GELVSIGGLNPDVNKKLSAGIASILESKLSIPKGRFYLKFYDSKRSDFGWNGTTF
S._bicolor_XP002441679        61 GELVSIGGLNPDVNKKLSAGIASILESKLSVPKSRFYLKFYDSKRSDFGWNGSTF
Z._mays_XP008677331           61 GELVSIGGLNPDVNKKLSAGIASILESKLSVPKSRFYLKFYDSKRSDFGWNGSTF
consensus                     61 *********....**.***....***.***..*.**.***.*.*.* *****.*.
```

Angiosperm MIF proteins with similarity to *AtMDL*3 (harboring a putative C-terminal PTS1 signal)

```
S._lycopersicum_XP004249685    1 MPCFNLSTNVNLDGVDTSDFFSEATKAVSSIIGKPENFVMVVLKGSVDISFG-GNKEPAA
V._vinifera_XP002264120        1 MPCVDISTNVNLEGVDADPIFSDVTKAVASIIGKPENYVMVLLKGSVAISFE-GNKEPAA
G._max_ACU19241                1 MPCLYITTNLNLDGVDTNPVFSEATTAVSTIIGKPEKFVMVILKSSVPISFE-GNKEPAA
L._japonicus_AFK35159          1 MPCLYIHTNINLDGVDTDSIFSEATTAVSTIIGKPEKFVMVLLKGSVPISFE-CNKEPAA
P._persica_XP007207503         1 MPCLYISTNVNLDGFDTDSIFSEATKAISSITGKPEDYVMVLLKGSVPISFGRSTSEPAA
A._thaliana_AtMDL3             1 MPCLYITTNVNFDGVNTDPFYSEVTKAVASIVGRPQNLVMVVLKGSVEIVFG-GNKEAAA
consensus                      1 ***....**.*..*.......*..*.*...*.*.*...***.**.**.*.*  ...*.**

S._lycopersicum_XP004249685   60 FAEIVSMGGINSDVKRKLIATLGGICQNRFSIPRTRFFLKVYDTTMATKFSKL
V._vinifera_XP002264120       60 FAEIISMGGINSEVKRKLIATIGTILQTKLSIPRTRFFLKVYDTTMAHKIAKL
G._max_ACU19241               60 YAEIVSMGGISTEVKRKLIATIGTILQSNLSIPRTRFFLKVFDVSAFRTNSKM
L._japonicus_AFK35159         60 YAEIISMGGINSEVKKKLIYTIGTILQSKLSIPRTRFFLKVFDTTLFRNKSKL
P._persica_XP007207503        61 YGELVAMGGINKPVKRQLIATLGTIMEAKLSIPKTRFFLKVVDISTATG-SKL
A._thaliana_AtMDL3            60 YAEIVSMGGITKQVKRELIATVGSILHTHFSIHPTRFIFKVFDINSLPLPSKL
consensus                     61 ..*...****...**..**.*.*.*.....**..***..**.*.....  .*.
```
