# Supplementary material for: Phylogeny and evolution of plant macrophage migration inhibitory factor/D-dopachrome tautomerase-like proteins
Source: BMC Evol Biol. 2015 Apr 14;15:64. doi: 10.1186/s12862-015-0337-x (PMC4407349; doi:10.1186/s12862-015-0337-x)
Supplement: Additional file 9: File S1. — MIF/MDL protein sequences used in this study. [file 12862_2015_337_MOESM9_ESM.pdf]

Supplemental File 1. MIF/MDL protein sequences used in this study.

>*A._thaliana_At*MDL1
MPTLNLFTNIPVDAVTCSDILKDATKAVAKIIGKPESYVMILLNSGVPIAFAGTEEPAAYGELIS
IGGLGPGVNGKLSETISEILQIKLSIDSSRFYIKFYDSPRPFFGYNGSTF

>*A._thaliana_At*MDL2
MPCLNLSTNVNLDGVDTSSILSEASSTVAKIIGKPENYVMIVLKGSVPMSFGGTEDPAAYGELVS
IGGLNADVNKKLSAAVSAILETKLSVPKSRFFLKFYDTKGSFFGWNGATL

>*A._thaliana_At*MDL3
MPCLYITTNVNFDGVNTDPFYSEVTKAVASIVGRPQNLVMVVLKGSVEIVFGGNKEAAAYAEIVS
MGGITKQVKRELIATVGSILHTHFSIHPTRFIFKVFDINSLPLPSKL

>*B._distachyon_*XP_003579042
MPCLNVSTNVNLDGVDTSAVLADASSAVATIIGKPEAYVMVVLKGSVPMAFGGTQEPAAYGELVS
IGGLSPDVNKKLSAGIAAILESKLSIPKSRFYLKFHDSKRSDFGWNGTTF

>*B._distachyon_*XP_003577817
MPQLSISTNVPVDAIVAADILKDCSRALAKIIGKPESYVMVSINGCVPTSFAGSEEPAAYGEIMS
IGGLGPGVNGKLSAALAEILETKLSISRSRFYVKFDDVQGYNLGFNGSTF

>*C._reinhardtii_*XP_001691775
MPTLNIITNVAGDRVTTSDVLKALSKAVASSVGKPEQWVMASVTTDKPMIYGGTEEPCAFGYLMS
IGSIGGDKNKKISAAICEVLTAKLGVPANRVYIQFSDAKASDVGWDGSTFA

>*G._max_*NP_001238163
MPTLDLFTNVPVDTVVASDILRDATKAVAKIIGKPESYVMILLNGGVPIAFAGTEEPAAYGELIS
IGGLGPSVNGKLSSTIAEILETKLYIDSSRFYIKFYDVQRSFFGFNGSTF

>*G._max_*NP_001236304
MPCLNLSTNVSLEGVDTSSILAEATSSVASIIGKPEAYVMIVLKGSVPIAFGGNEQPAAYGELVS
IGGLNPSVNKELSAAIASILETKLSVPKSRFFLKFYDTKGSNFGWNGSTF

>*G._max_*NP_001237629
MPCLNLSTNVNLDGIDTSSILSEATSTVASIIGKPEAYVMIVLKGSVPISHGGSEQPAAYGELVS
IGGLSPDVNKKLSAGIASILENKLSVPKSRFYLKFYDTKGSNFGWNGSTF

>*G._max_*ACU19241
MPCLYITTNLNLDGVDTNPVFSEATTAVSTIIGKPEKFVMVILKSSVPISFEGNKEPAAYAEIVS
MGGISTEVKRKLIATIGTILQSNLSIPRTRFFLKVFDVSAFRTNSKM

>*H._sapiens_Hs*MIF
MPMFIVNTNVPRASVPDGFLSELTQQLAQATGKPPQYIAVHVVPDQLMAFGGSSEPCALCSLHSI
GKIGGAQNRSYSKLLCGLLAERLRISPDRVYINYYDMNAANVGWNNSTFA

>*H._sapiens_Hs*DDT

MPFLELDTNLPANRVPAGLEKRLCAAAASILGKPADRVNVTVRPGLAMALSGSTEPCAQLSISSI
GVVGTAEDNRSHSAHFFEFLTKELALGQDRILIRFFPLESWQIGKIGTVMTFL

>*H._vulgare*_BAJ92045
MPCLNVSTNVNLEGVDTSAVLADASSTVATIIGKPEAYVMVVLKGSVPMAFGGTQEPAAYGELVS
IGGLNPDVNKKLSAGIASILESKLSISKSRFYLKFHDSKRSDFGWNGTTF

>*H._vulgare*_BAJ88384
MPQLSLSTNVPVDAVVAADILRDCSRALARIIGKPESYVTVSIDGSVPTSFAGSEEPAAYGEIMS
IGGLGPGVNGKLSAALADILEAKLSISPSRFYVKFDDVQGYNVGFNGTTF

>*L._japonicus*_AFK35159
MPCLYIHTNINLDGVDTDSIFSEATTAVSTIIGKPEKFVMVLLKGSVPISFECNKEPAAYAEIIS
MGGINSEVKKKLIYTIGTILQSKLSIPRTRFFLKVFDTTLFRNKSKL

>*L._japonicus*_AFK37368
MPCLNLSTNVNLDGVDTSSILSEATSTVATLIGKPEAYVMIVLKGSVPVSFGGTEQPAAYGELVS
IGGLNPDVNKKLSAAIASILETKLSVPKSRFFLKFYDTKGSNFGWNGSTF

>*L._japonicus*_AFK37854
MPTLNLFTNIPVDAVVASDILRDATKAVAKIIGKPESYVMILLNGGVPIAFGGTEEPAAYGELIS
IGGLGPSVNGKLSSTIAEILQTKLYIDGSRFYIKFYDVQRSFFGFNGSTF

>*O._sativa*_NP_001058472
MPQLSLTTNVPVDAVVAADIIKDCSKALARIIGKPESYVMVSISGSVPMSFAASEEPAAYGELMS
IGGIGPGVNGKLSAALAEILETKLSVSRSRFYVKFDDVKGFNLGFNGSTF

>*O._sativa*_ABG22330
MPCLNVSTNVNLDGVDTSAVLADASKTVATIIGKPEAYVMVVLKGSVPMAFGGTQEPAAYGELVS
IGGLNPDVNKKLSAGIASILESKLSIPKGRFYLKFYDSKRSDFGWNGTTF

>*P._patens*_XP_001768921
MPTLNIQTNVPLDGVVTSDILKDASKAVAQILSKPESYVLISLRGGIPMSFGGTEEPAAYGELIS
IGGVGPDTNKRLSAAISDILKTKLSVPSNRFYIKFFDVKRSDMGWNGSTF

>*P._persica*_XP_007207503
MPCLYISTNVNLDGFDTDSIFSEATKAISSITGKPEDYVMVLLKGSVPISFGRSTSEPAAYGELV
AMGGINKPVKRQLIATLGTIMEAKLSIPKTRFFLKVVDISTATGSKL

>*P._persica*_XP_007207497
MPCLNISANVSLEGVDTSSILSEATSTVAKIISKPEAYVMIVLKGSVPIAFGGTEQPAAYGELVS
IGGLNPDVNKKLSAAIAAILETKLSVPKSRFFLKFYDTKGSNFGWNGSTF

>*P._persica*_XP_007225890
MPTLNLFTNLPVDAVVASDILKDATKAVSKIIGKPESYVMILLNGSVPMAFAGTEEPAAYGELIS
IGGIGPSVNGKLSSTIAEILETKLSIDSSRFYIKFYDVERPFFGFNGSTF

>*P._sitchensis*_ABK23267
MPTLNLSTNVPVDSVVSSDILKDASKSVARIIGKPESYVMVLLKGGVPMLFGGSEEPAAYGEVVS
IGGLGPGVNGKLSAAIADILESKLSVDKSRFYIKFYDVEGSYFGFRGSTF

>*P._sitchensis*_ABK23881
MPSLNISTNVPLEGLNTSEILSETSKSVAKIIGKPEAYVMVQLKGSVAISFGGTEEPAAYGELVS
IGGLGSDTNKKLSAAIATILDTKLKVPKSRFYIKFYDVKRSDFGWNGTTF

>*S._bicolor*_XP_002438947
MPTLNLRTNVPVDAVVAADILKDCSKAVARIIGKPESYVMVSINGSVPMSFAGSEEPAAYGELVS
IGGIGPGVNGKLSAAVAEVLETKLSVSRSRFYIKFDDVQRSNFGFNGSTF

>*S._bicolor*_XP_002441679
MPCLNVSTNVNLEGVDTSVILAEASKSVANIIGKPEAYVMVVLKGSVPMAFGGTQEPAAYGELVS
IGGLNPDVNKKLSAGIASILESKLSVPKSRFYLKFYDSKRSDFGWNGSTF

>*S._lycopersicum*_XP_004242799
MPTLNLFTNLPVDAVIASDILKDATKAVAKIIGKPESYVMILLNGGVPIAFAGTEAPAAYGELIS
IGGLGPSVNGKLSSTIAEILQTKLSIDSDRFYIKFYDSPRPFFGFNGSTF

>*S._lycopersicum*_XP_004249062
MPCLNISTNVNLEGVDTSSVLSEATSTVAKLIGKPEAYVMIVLKGSVPMAFGGTEQPAAYGELVS
IGGLNADVNKKLSAAIADILETKLSIPKSRFFLKFYDTKGSFFGWNGSTF

>*S._lycopersicum*_XP_004249685
MPCFNLSTNVNLDGVDTSDFFSEATKAVSSIIGKPENFVMVVLKGSVDISFGGNKEPAAFAEIVS
MGGINSDVKRKLIATLGGICQNRFSIPRTRFFLKVYDTTMATKFSKL

>*S._moellendorffii*_XP_002961952
MPTLNLSTNVPADSIVSSDILKDASKAVSRIIGKPEQYVMIVLKDSVPMSFGGTEEPAAYGEVIS
IGGLGPSVNKELSEALSEILESKLSVPPSRFYIKFYDVQRSFFGWNGSTF

>*S._moellendorffii*_XP_002966661
MPTLNISTNVPLDGVSTSDILKDASRTVARVLGKPESYVMIIINGAVPISFGGSEEPAAYGELVS
IGAISPDSNKKLSKAIAELLQSKLAVPPNRFYIKFYDVKGSNFGWNGSTF

>*S._moellendorffii*_XP_002964007
MPILTISTNTSVDSSTSFYILQEATAAVARVLGKPESSMMVLLNDRVPILFGGSREAAAYGELVS
IGAIAPDSNRKVSKALAGILESRLSVPPSRFYIKFYDVKGSNVGYNGSTY

>*S._moellendorffii*_XP_002983015
MPVLTIHTNVVLLDGSLMSSVVSKLSHEVAKTTGKPESYVMVLLHGGVTLAFQGSHESAAAYGEL
VSIGGLSPGVNQDLCKAIARVLEEELKVPPSRCYIKFYQEQACVLRIWCSVSLFLFDEILSF

>*V._carteri*_XP_002955179
MPTLNVITNVPCDRVTSSDVLKALSKAVSKSVGKPEQWVMCSLTTDKPMIYGGSEEPCAFGYFMS
IGAIGGDVNQPNIRQISAAICEVLSTHLGVPAARVYIEFSDVNASDVGWNGSTFV

>*V._vinifera*_XP_002264120
MPCVDISTNVNLEGVDADPIFSDVTKAVASIIGKPENYVMVLLKGSVAISFEGNKEPAAFAEIIS
MGGINSEVKRKLIATIGTILQTKLSIPRTRFFLKVYDTTMAHKIAKL

>*V._vinifera*_XP_002263560
MPCLNLSTNVSLDGVDTSSILSEATSTVAKIIGKPEAYVMIVLKGSVPIAFGGTEQPAAYGELVS
IGGLNPGTNKELSAAISAILETKLSVPKSRFFLKFYDSKGSNFGWNGSTF

>*V._vinifera*_XP_002264373
MPTLNLFTNVPVDAVVASDILKDCTKAVAKIIGKPESYVMILLNGGVPIEFAGTEEPAAYGELIS
IGGLGPSVNGKLSSTIAEIIQTKLSIDSARFYVKFYDVERSFFGFNGSTF

>*Z._mays*_XP_008677331
MPCLNVSTNVNLEGVDTSAILAEASKSVANIIGKPEAYVMVVLKGSVPMAFGGTQEPAAYGELVS
IGGLNPDVNKKLSAGIASILESKLSVPKSRFYLKFYDSKRSDFGWNGSTF

>*Z._mays*_NP_001150913
MPTLNLSTNVPVDAVVAADILRDCSKAVARIIGKPESYVMVSVNGSVPMSFAASEEPAAYGELVS
IGGIGPGVNGKLSAAVAEVLEAKLSVSRSRFYIKFDDVRGHNFGFNGSTF
